# Water‐Mediated Phosphoryl Wires Stabilize Pathological Tau Fibrils

**DOI:** 10.1002/anie.202521499

**Published:** 2026-05-23

**Authors:** Lokeswara Rao Potnuru, Austin DuBose, Fiona Mon, Mesopotamia S. Nowotarski, Michael Vigers, Boqin Zhang, Chung‐Ta Han, John E. Straub, Songi Han

**Affiliations:** ^1^ Department of Chemistry Northwestern University Evanston Illinois USA; ^2^ Department of Chemistry and Biochemistry University of California Santa Barbara California USA; ^3^ Department of Chemistry Boston University Boston Massachusetts USA; ^4^ Department of Chemical Engineering University of California Santa Barbara California USA

**Keywords:** ^31^P solid‐state NMR, DNP, molecular dynamic simulations, multiple‐quantum spin counting, phosphorylation, tauopathy

## Abstract

Hyperphosphorylation of tau is a hallmark of tauopathies, with specific phosphorylation sites elevated in pathological fibrils. However, the molecular role of this post‐translational modification (PTM) in driving tau aggregation remains unclear. In‐register fibril assembly places phosphoryl groups on adjacent monomers at ∼4.8 Å spacing, requiring an energetically favorable arrangement. Conventional intuition holds that closely packed phosphoryl groups should be electrostatically unfavorable. We test the opposing hypothesis: that phosphoryl groups within the fibril core associate into an extended “wire” that stabilizes the amyloid fibril. We examined two phosphorylation sites linked to neurodegeneration, serine 305 (S305^p^) and tyrosine 310 (Y310^p^), using seeding‐competent fibrils of the tau peptide jR2R3‐P301L. Multiple‐quantum spin counting (MQ‐SC) by ^3^ ^1^P solid‐state NMR with dynamic nuclear polarization (DNP) revealed at least six phosphorus spins linearly arranged within a protofibril, consistent with a MQ coherence order of four. Molecular dynamics simulations identified water‐mediated phosphoryl wire geometries, and 2D ^1^H–^3^ ^1^P heteronuclear correlation NMR confirmed water‐bridged phosphoryl‐phosphoryl contacts. Denaturation experiments showed that S305 phosphorylation increased fibril stability relative to the unmodified peptide. These findings show that phosphorylation within the tau fibril core promotes fibril registry and stability through water‐mediated, hydrogen‐bonded phosphoryl wires, which may be a structural signature for next‐generation pathological tau binders.

## Graphical abstract

Extended 1D phosphoryl “wires” stabilize in‐register amyloid tau fibrils, as demonstrated by multiple‐quantum spin‐counting NMR, TEM, and MD simulations, using fibrils of tau peptide jR2R3‐P301L (tau295–313) with phosphorylation at S305 or Y310.

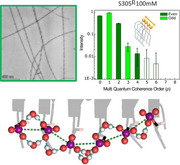

## 1 Introduction

Tau is an intrinsically disordered protein (IDP) that serves many biological functions, most notably as a scaffold for stabilizing microtubules in neurons. Under physiological conditions, hyperphosphorylation of tau is proposed to play an essential role in regulating tau function and/or inducing aggregation of free tau, which is a hallmark of pathogenic conditions [1, 2]. The human tau protein isoforms report on phosphorylation at 40–50 different sites out of 85 sites of serine, threonine, and tyrosine residues under pathological conditions [1]. However, neither the abundance nor the combinations of post‐translational modification (PTM) that occur on a given tau molecule are known, nor their molecular‐level roles. Hyperphosphorylated tau proteins are key constituents of neurofibrillary tangles (NFTs) [2] found under several neurodegenerative disease conditions referred to as tauopathies, including Alzheimer's Disease (AD) [3], Chronic Traumatic Encephalopathy (CTE) [4, 5], Pick's Disease (PiD), Corticobasal Degeneration (CBD) [6, 7], Progressive Supranuclear Palsy (PSP) [8], and Argyrophilic Grain Disease (AGD) [9, 10]. While the current understanding of neuropathology is that the hyperphosphorylated tau and its fibrillar assemblies are neurotoxic forms of tau, it remains unclear whether hyperphosphorylation is an aggravating factor in, or a reflection and consequence of, the cellular and molecular processes underlying tauopathies and pathological tau self‐assembly [11, 12]. Research suggests that phosphorylation occurs before aggregation [1] and that phosphorylation at different sites has different effects on biological processes and pathogenic developments [2, 11]. Hyperphosphorylation of soluble tau may contribute to neurodegeneration by weakening the association of tau with microtubules and destabilizing microtubules, or by increasing the pool of free intracellular tau available for aggregation [12, 13, 14, 15]. While there may be downstream biological consequences of tau phosphorylation, this study addresses an important unknown in the field, namely, whether phosphorylation has direct biophysical consequences on filament formation and the stability of phosphorylated tau proteins [12, 14]. Resolving this question may be key to the synthetic replication of tau NFTs with the molecularly defined folds and quaternary structures that serve as diagnostic hallmarks of different tauopathies, including AD, CTE, CBD, PSP, AGD, and other tauopathies. This is especially relevant given that PTM patterns of tau appear to differ between tauopathies [16, 17].

We recently reported that phosphate‐containing molecules, including dissolved monophosphates and ATP, form dynamic assemblies in equilibrium with their monomeric forms across a wide range of biological conditions in aqueous solution [18]. This raised the possibility of a previously unrecognized role for phosphate‐containing and phosphorylated residues in facilitating self‐assembly and fibril stabilization. Irrespective of whether hydrogen bonding between phosphoryl groups constitutes a sufficiently dominant driving force for aggregation, once tau fibrils are formed, phosphoryl groups should be a major stabilizing factor for fibrils once they form extended, water‐mediated, hydrogen‐bonded networks. At the minimum, adjacent phosphoryl groups along the fibril elongating axis may not be destabilizing despite their net negative charge at close spacing (within 4.8 Å) to adjacent phosphoryl groups in β sheet arrangements, owing to water‐mediated hydrogen bonding strengthened by delocalized partial charges on the oxygen atoms [19]. Next, direct experimental evidence is needed to determine whether phosphoryl groups on tau associate into a stabilizing network with long‐range order within tau filaments.

Cryogenic electron microscopy (cryo‐EM) has been transformative in elucidating the structure of tau fibrils from the postmortem brain of tauopathy patients, while solution and solid‐state NMR (SSNMR) spectroscopy elucidated valuable structural information on pathological tau protein [20, 21, 22]. Tau exhibits enhanced phosphorylation under pathological conditions at several serine sites (e.g., S202, S262, S356, and S396), while phosphorylation of threonine, Thr181, Thr217, and Thr231, is emerging as a promising diagnostic signature of AD. However, cryo‐EM structures of tau fibrils do not reveal atomic‐level details about the occurrence or organization of phosphorylated residues, despite antibody evidence for their presence [23, 24]. This limitation arises from insufficient resolution, the heterogeneous nature of phosphorylated species, and/or the frequent localization of these modifications outside the fibril core. length tau modified by T8‐3E (S202E, T205E, S208E) forms a fibril core extending from R3 to the C terminus, whereas full‐length tau modified by PHF1‐4E (S396E, S400E, T403E, S404E) exhibits a core spanning R2–R4. Cryo‐EM resolves only the rigid fibril core and therefore cannot access the structural consequences of phosphorylation that lie outside this region. Although NMR can capture signals from the dynamic fuzzy coat, complete high‐resolution structure determination of pathological tau fibrils by NMR remains rare. Pseudophosphorylation studies have shown that full‐length tau modified by T8‐3E (S202E, T205E, S208E) forms a fibril core extending from R3 to the C terminus, whereas full‐length tau modified by PHF1‐4E (S396E, S400E, T403E, S404E) exhibits a core spanning R2–R4 [25]. However, most of these studies employ pseudophosphorylation [26, 27] and therefore do not inform on the effects of actual phosphoryl groups on tau assembly.

This distinction is consequential if the role of phosphoryl groups extends beyond electrostatic interactions to the formation of an extended hydrogen‐bonded assembly along the fibril axis. A recent study found that GSK3β‐phosphorylated 2N4R tau exhibits enhanced liquid‐liquid phase separation (LLPS) and forms a fibril core with a C‐shaped fold similar to that observed in AD [11]. Solution state NMR has further reported effects of phosphorylation on transient local secondary structures and the global conformational ensemble of modified tau [28, 29, 30, 31], with certain phosphorylation combinations in the polyproline rich region (PRR) shown to induce secondary structural changes [29, 30]. Phosphorylation at specific PRR residues, for example S202 and T205, alters the conformational ensemble of tau monomers and their aggregation propensity [32, 33], while phosphorylation of S262 in the R1 repeat domain weakens tau‐microtubule association [28, 34, 35]. Despite extensive interest in the role of phosphorylation in pathological tau aggregation, the biophysical mechanism by which phosphorylation facilitates intermolecular tau association and stabilizes the fibril core is a new concept and remains unexplored.

This study uses SSNMR augmented by dynamic nuclear polarization (DNP) to capture through‐space ^31^P–^31^P dipolar couplings to determine whether extended phosphoryl group assemblies are present in tau fibrils. Because the ^31^P NMR spectral signature of individual phosphoryl species is indistinguishable from one another, we employ multiple‐quantum (MQ) spin counting (SC) to determine the coherence order of coupled spins. ^31^P MQ‐NMR has previously been used to establish connectivity between ^31^P nuclei in networks of solid phosphate clusters and crystals [36, 37]. Tycko applied ^13^C MQ‐SC to powder samples of amyloid fibrils composed of seven‐residue Aβ_16‐22_ fragments or full‐length Aβ_1‐40_ to probe the supramolecular organization of β‐sheets, observing MQCO up to 4. They concluded that at least seven Aβ_16‐22_ fragments stack in an antiparallel β‐sheet arrangement, while full‐length Aβ_1‐40_ forms in‐register structure(s) [38, 39]. We recently extended ^31^P MQ‐SC analysis from solid samples to small clusters in solution state upon vitrification, combined with DNP‐enhanced NMR signal amplification, to capture assemblies formed under native solution conditions [40]. Here, we apply ^31^P MQ‐SC to vitrified fibrils of phosphorylated tau fragments to determine whether phosphoryl groups organize into extended architectures along the fibril axis that contribute to fibril stabilization.

We used a critical tau fragment spanning D295 to V313 of 4R tau, which spans the junction between the R2 and R3 repeat domains and contains the P301L mutation (jR2R3‐P301L). This peptide forms fibrils adopting pathological folds and displaying prion‐like seeding properties [41, 42]. Two sites within jR2R3‐P301L have been reported by proteomics studies to be phosphorylated under pathological conditions: S305 and Y310 [17]. We hence prepared phosphorylated (not pseudo‐phosphorylated) jR2R3‐P301L, at site S305 or Y310, one at a time, referred to as S305^p^ and Y310^p^. These peptides are prepared at natural isotopic abundance for all nuclei. The study commenced by assessing the quality and stability of the phosphorylated jR2R3‐P301L tau peptide fibril by thioflavin T fluorescence (ThT) assay and negative‐stain transmission electron microscopy (TEM). We proceeded with ^31^P SSNMR MQ‐SC to investigate whether the phosphoryl groups on specific sites of the peptide form a higher‐order “wire”‐like network. Molecular dynamics simulations of phosphorylated fibrils in explicit water were conducted to identify energetically stable and structurally plausible geometries of extended phosphoryl assemblies. Quantum mechanical simulations of ^31^P MQ‐SC NMR for spin systems with geometries guided by MD‐derived structures and prototype paired helical filaments were performed to analyze the experimental ^31^P MQ‐SC results and determine the minimal number of defect‐free, in‐register stacked phosphoryl groups in tau fibrils. The analysis leverages the established cryo‐EM results showing that tau proteins stack in parallel, in‐register fashion [42]. Finally, 2D ^1^H–^31^P HETCOR experiments were performed on phosphorylated fibrils to obtain spectroscopic evidence for bridging water molecules that mediate fibril stabilization.

## 2 Results and Discussion

### 2.1 Aggregation Assays

**FIGURE 1:**
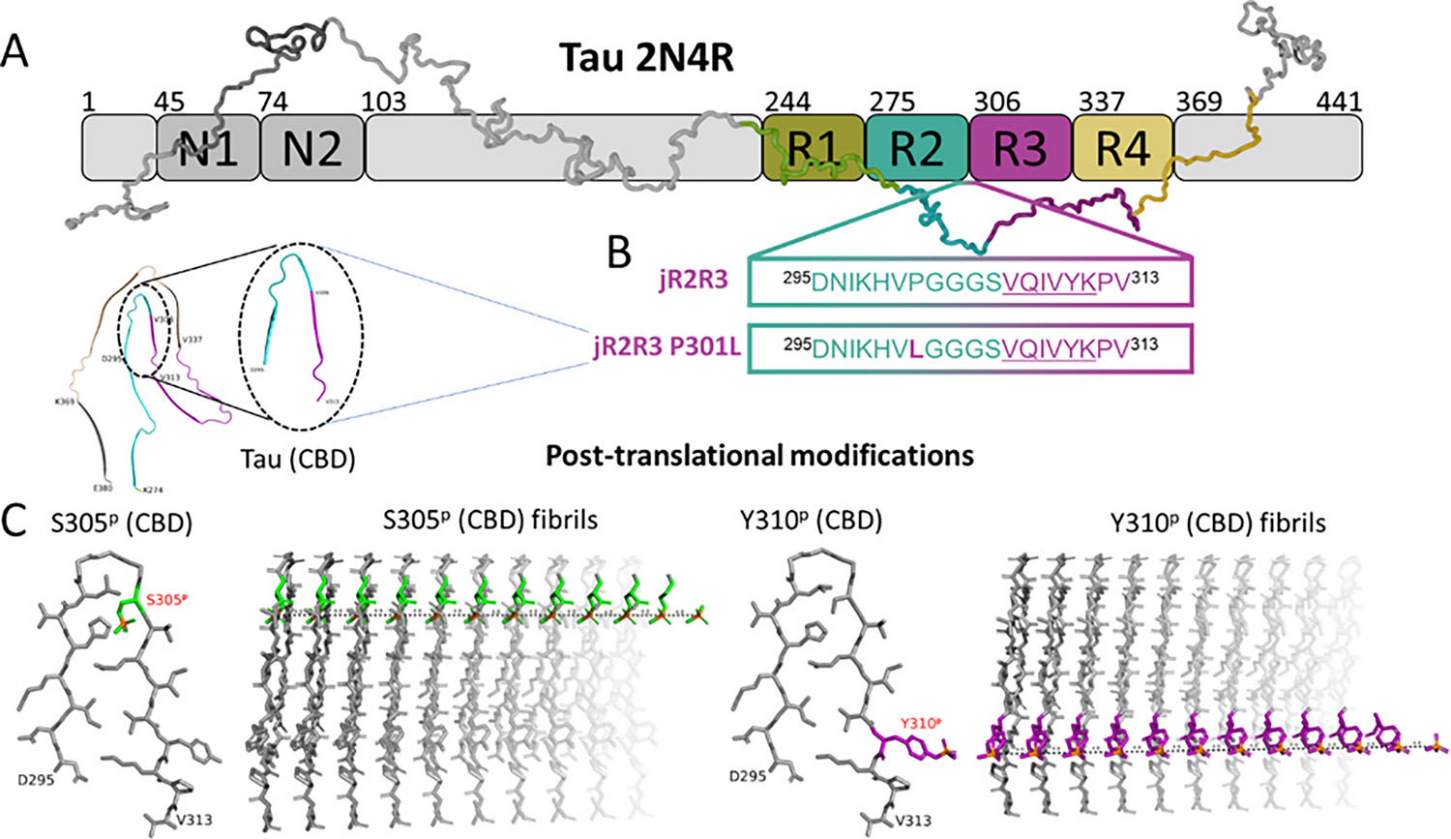
(A) Schematic representation showing an overview of the tau construct 2N4R. The elements are color‐coded for clarity with R1 highlighted in Olive, R2 in Teal, R3 in Purple, and R4 in Sand. PDB structure representing 4R tauopathies CBD with PDB ID #6VH7 [7] (B) Strand‐loop‐strand forming peptide segments designated as jR2R3 and jR2R3 P301L are displayed with the PHF6 sequence underlined, and residues mutated relative to jR2R3 shown in bold. jR2R3/jR2R3 P301L sequences represent the R2/R3 splice junction of tau spanning from D295 to V313. (C) Post‐translational modifications (PTMs) of jR2R3 P301L are presented in a hypothetical CBD configuration. Serine 305 (Green) and Tyrosine 310 (Purple) are the residues at which the phosphoryl group is added. Stacking of the monomers is proposed as displayed in the above figure caption.

Figure 1 depicts the 19‐residue jR2R3 peptide sequence (D295–V313) spanning the R2–R3 junction of the 4R tau isoform, and phosphorylation at S305 and Y310, illustrated in a hypothetical CBD structure [7], with the monomer stacked in‐register and parallel along the fibril axis.

**FIGURE 2:**
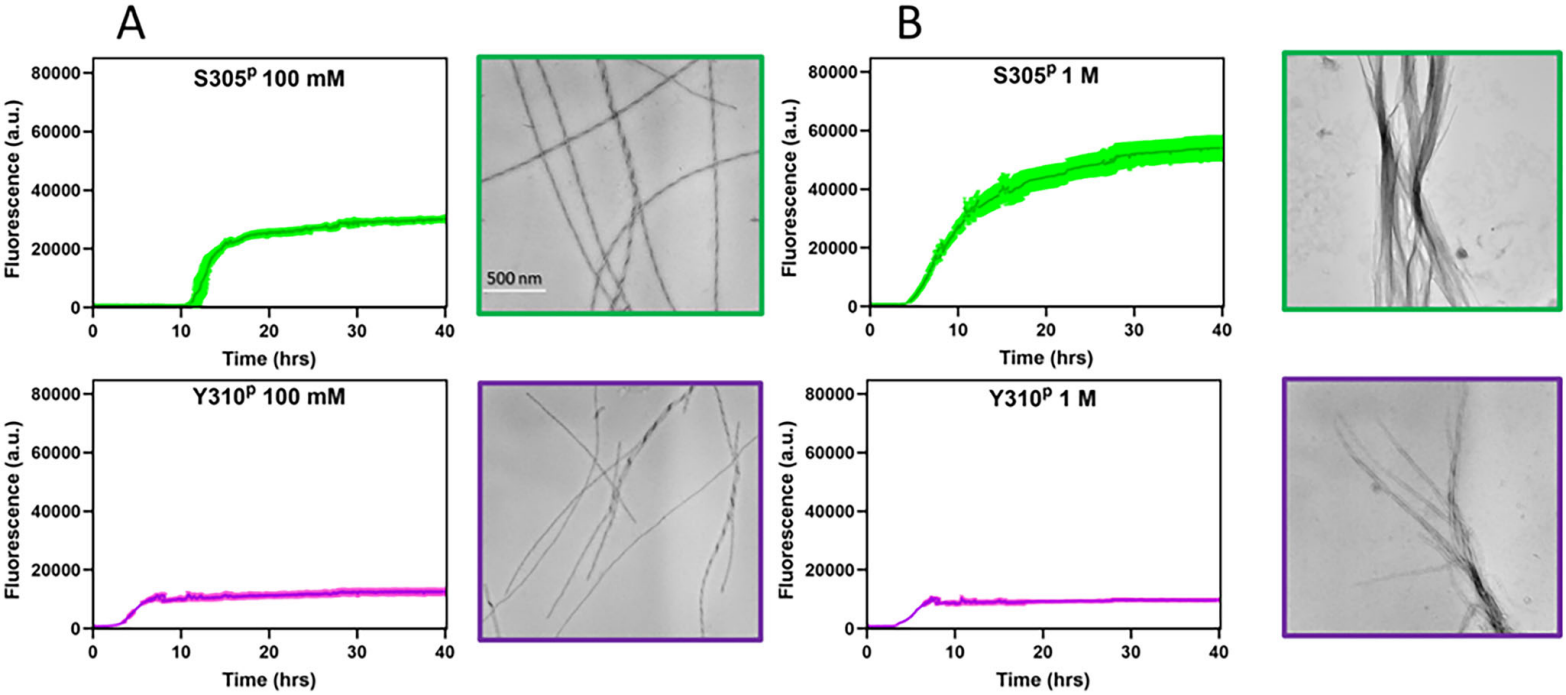
Monitoring the fibril aggregation of S305^p^ (Green) and Y310^p^ (Purple) jR2R3 P301L fibrils using the Thioflavin T (ThT) fluorescence assays and TEM micrographs for (A) 100 mM NaCl and (B) 1 M NaCl conditions, respectively. The scale bar is uniform for all TEM images.

The S305^p^ and Y310^p^ fibrils were prepared as described in Materials and Methods (Section S1.1), with the addition of heparin in a solution of 100 mM or 1 M NaCl. High salt concentration screens electrostatic interactions and sequesters water away from the protein, thereby amplifying the effect of hydrophobic association of tau. The high salt concentration is not physiologically relevant, but the effect of such conditions on the protein can nonetheless mimic physiologically relevant conditions, mimicking electrostatic screening, crowding, and/or enhanced hydrophobic effects [43]. A ThT assay was used to monitor fibril formation and stability under different salt concentrations (see Section S1.2 for Materials and Methods). Both S305^p^ and Y310^p^ fibrils produced strong ThT fluorescence signals (Figure 2), indicative of robust β‐sheet formation. S305^p^ fibrils yielded higher fluorescence than Y310^p^ fibrils under both salt concentrations. The morphology of S305^p^ and Y310^p^ fibrils was further examined by negative stain transmission electron microscopy (TEM): S305^p^ fibrils were more abundant and exhibited greater structural definition, including paired helical filaments and ribbon‐like structures, compared to Y310^p^ fibrils, which displayed fewer distinct morphological features (Figure 2).

### 2.2 Stability Revealed by Fibril Denaturation Experiments

The stability of these fibrils was tested using guanidinium hydrochloride (GdnHCl) at varying concentrations from 0 to 1 M. GdnHCl is a denaturant that can disrupt the natural structure of proteins by breaking hydrogen bonds and disturbing β‐sheet secondary structures [44]. ThT fluorescence was measured after equilibrating the samples in GdnHCl at different concentrations for 24 h. Fibrils that withstand denaturation under more severe conditions are considered more stable. Therefore, a greater decrease in ThT fluorescence with increasing GdnHCl concentration indicates lower fibril stability.

**FIGURE 3:**
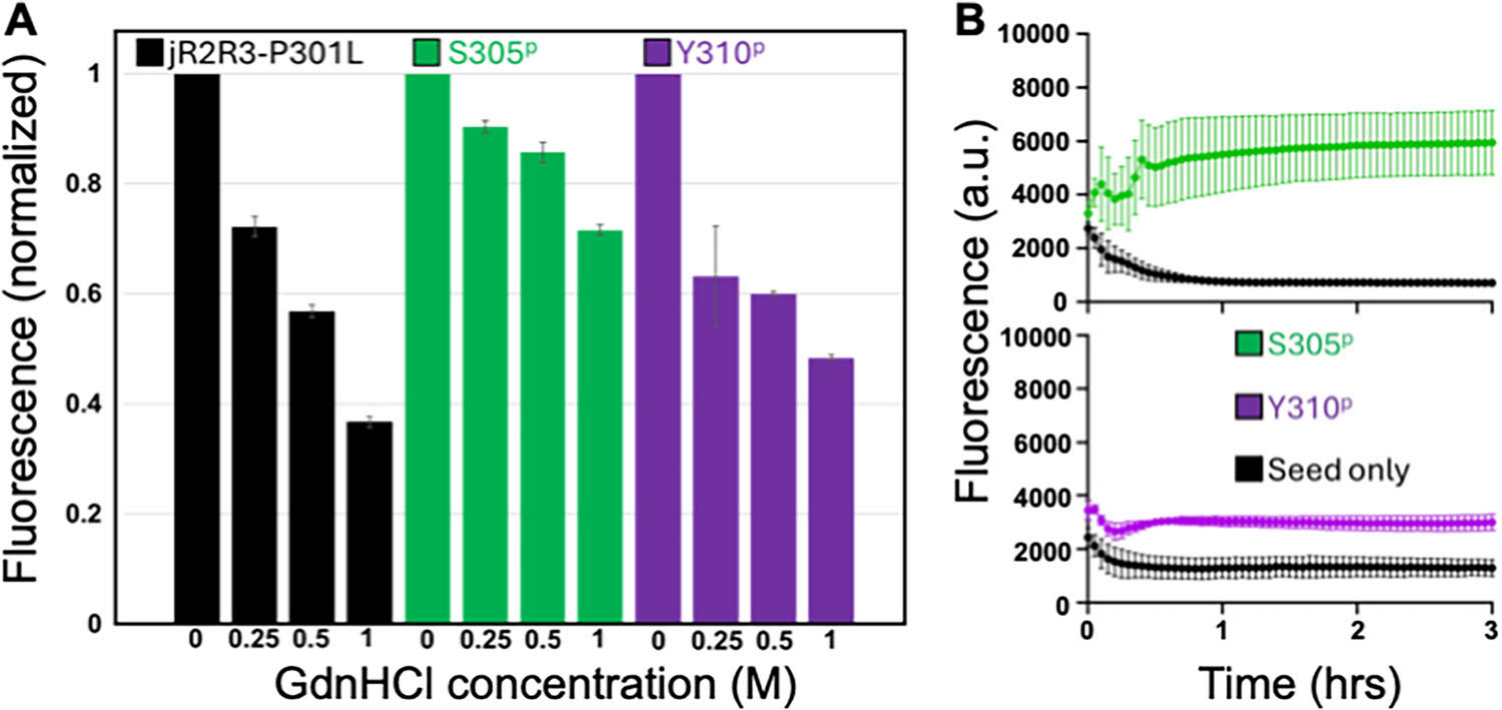
(A) Fibril denaturation experiment on fibrils prepared with 100 mM NaCl conditions, normalized ThT fluorescence as a function of Guanidinium hydrochloride (GdnHCl) concentration after 24 h of incubation at a temperature of 37°C. S305^p^ retains the most fluorescence at the highest concentration of GdnHCl, indicating a more stable fibril compared to both jR2R3‐P301L and Y310^p^. (B) ThT fluorescence from the seeding experiment, where equal amounts of each seed (black) were added to their corresponding monomer type, shows that S305^p^ has the highest increase in fluorescence, indicating more fibril formation compared to Y310^p^.

The stability of the jR2R3 P301L fibrils, prepared under 100 mM NaCl conditions, measured using the GdnHCl assay, is shown in the Figures 3A and S15. To standardize the results, all values were compared to the initial fluorescence of the corresponding fibrils before denaturation. Under denaturing conditions with GdnHCl, all peptide fibrils exhibited reduced fluorescence intensity, indicating that the fibrils at least partially disassemble. Notably, at 1 M GdnHCl concentration, the peptide fibrils (prepared under 100 mM NaCl conditions) displayed distinct differences in stability between S305^p^ and Y310^p^. S305^p^ fibrils show a net loss of 28% ± 0.96% and Y310^p^ a net loss of 52% ± 0.57% in fluorescence, whereas jR2R3 P301L fibrils without any phosphorylation showed the greatest net loss of 63% ± 0.97%. The TEM images (Figure S15) track the characteristic features seen from denaturation: greater fragmentation is observed in the jR2R3 P301L fibril compared to S305^p^ or Y310^p^ fibrils with increasing GdnHCl concentration. These results show that phosphorylated jR2R3 fibrils are more stable compared to non‐phosphorylated jR2R3 fibrils, with the S305^p^ fibrils exhibiting the greatest stability. These results suggest higher‐order associations stabilize the phosphorylated fibril.

### 2.3 Seeding Competency Studies

We next test the hypothesis that fibrils with neater packing and greater stability are more seeding competent, given that templating of naïve tau requires it to adopt the same structural folding as the tau folded within the fibril seed. Using the ThT fluorescence amplitude as a guide to assess seed performance, we found that S305^p^ fibrils are more competent in seeding than Y310^p^ fibrils (Figure 3B). This finding is consistent with the earlier discovery that S305^p^ fibrils are more stable than Y310^p^ fibrils.

### 2.4 SSNMR MAS DNP Studies of Vitrified Fibrils

**FIGURE 4:**
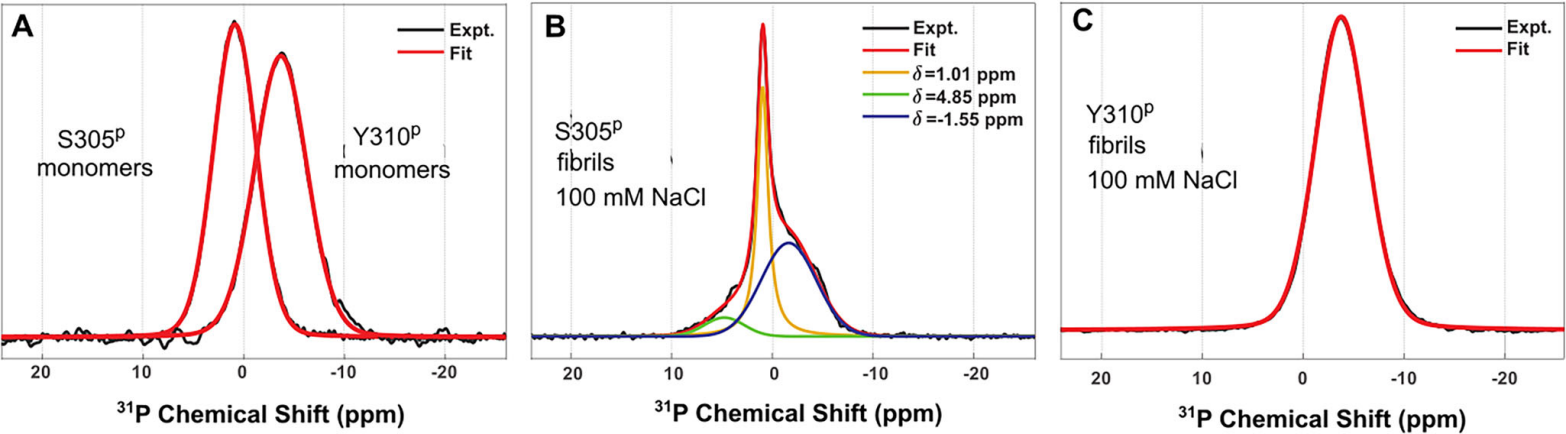
All spectral data were obtained at 10 kHz MAS frequency and 100 K temperature under DNP. A signal enhancement of 40‐fold was attained with DNP for each spectrum. Further details are given in SI. (A–C) ^31^P NMR spectra of S305^p^ jR2R3 P301L and Y310^p^ jR2R3 P301L samples and the corresponding deconvolution of the spectra using DMfit software. (A) Overlay of monomer spectra of S305^p^ and Y310^p^, (B) S305^p^ fibril formed in 100 mM NaCl, (C) Y310^p^ fibril formed in 100 mM NaCl, respectively.

SSNMR studies were performed under MAS frequency of 10 kHz and with DNP at 100 K on S305^p^ and Y310^p^ fibril samples at natural isotopic abundance for ^13^C (and all nuclei). First, one‐dimensional (1D) ^31^P NMR spectra by ^1^H‐^31^P cross‐polarization (CP) were recorded. The ^31^P NMR spectra of vitrified monomers of both S305^p^ and Y310^p^ show a broad Gaussian lineshape (Figure 4A), a characteristic of structural heterogeneity or disorder. In contrast, the ^31^P NMR spectra of the S305^p^ fibrils prepared in 100 mM or 1 M NaCl solution show a dominant (41%) spectral component with a significantly narrowed line (Figure 4B) compared to that of the monomer (Figure 4A). This result implies that the phosphoryl groups in S305^p^ experience a higher degree of structural homogeneity in fibrils than in monomers, as well as compared to Y310^p^ fibrils and monomers. However, a spectral decomposition analysis is needed to dissect key details.

The ^31^P NMR spectra were analyzed using a Gaussian/Lorentzian model in DMfit software (shown in Figures 4A–C, and S2A,B, and Tables S1 and S2) [45]. The monomer spectra of S305^p^ and Y310^p^ fit to a Gaussian lineshape, centered at 0.9 and −3.9 ppm, with a full width at half maximum (FWHM) of 800 and 960 Hz, respectively. Deconvolution of the ^31^P spectra of the S305^p^ fibrils, prepared in either 100 mM or 1 M salt solution, resulted in three distinct spectral components centered around 4.85, 1.0, and −1.55 ppm with FWHM of 750, 210, and 1040 Hz, respectively. These components may represent three physically distinct phosphoryl group populations. The narrowest spectral component centered at a chemical shift of 1.0 ppm is the dominant component (41%), adopting a Lorentzian line with a FWHM of 210 Hz—this significant linewidth reduction is a characteristic of structural homogeneity in ^31^P environments. In contrast, the other two are broader Gaussian components, one centered at 4.85 ppm (750 Hz, 8%) and another at −1.55 ppm (1040 Hz, 51%), that we attribute to fibril populations with alternative conformers, imperfect alignments, or amorphous aggregates. Notably, the ^31^P NMR spectra of the Y310^p^ fibrils, prepared in either 100 mM or 1 M salt solution, and their monomers showed no spectral component with a narrow Lorentzian component.

Line broadening in NMR arises from both homogeneous and inhomogeneous contributions. The homogeneous linewidth can be obtained from spin‐relaxation times (T_2_), measured by cross‐polarization (CP) followed by a Hahn‐echo from $\frac{1\;}{\left(\pi T_{2}\right)}$, as listed in Table S3. The inhomogeneous line width contribution for each sample was then obtained by subtracting the homogeneous spectral component from the total observed spectral line (Table S4). The linewidth of the earlier discussed Lorentzian narrow component of S305^p^ (211 Hz) is fully accounted for by homogeneous broadening. In contrast, the inhomogeneous linewidths for the broader signals were in the range of 550–700 Hz across all samples. The homogeneous linewidth contributions across all samples were consistently in the range of 210–280 Hz, except for the broad component (1040 Hz) of S305^p^ fibrils that shows a homogeneous broadening of 410 Hz (Table S4). We conclude that the homogeneous broadening is comparable across samples, and the major difference in the line widths across all samples arises from inhomogeneous broadening, attributed to chemical shift differences caused by structural heterogeneity or disorder.

Homogeneous broadening arises from both coherent and incoherent interactions. Coherent interactions include CSA, as well as homo‐ and heteronuclear dipolar couplings for spin ½. Since the spectra were recorded at a MAS rate of 10 kHz and with proton decoupling applied, these anisotropic contributions were largely averaged out owing to small magnitudes for homonuclear dipolar coupling (180 Hz for a ^31^P–^31^P distance of 4.8 Å) and heteronuclear dipolar coupling (1000 Hz for a ^1^H‐^31^P distance of 2 Å) relative to the MAS rate. The CSA, determined by the sideband amplitude analysis of the MAS spectrum, was found to be 80–100 ppm (13–16 kHz) for all samples (see Table S5); however, the observed linewidths are not broadened by CSA, since this interaction is also averaged out under MAS. As expected, the simulated 1D ^31^P NMR spectra for the relevant geometries (Figure 7) show indistinguishable linewidth for different dipolar couplings, given the small inter‐^31^P dipolar couplings relative to the MAS frequency (Figure S10). In summary, the only source of homogeneous broadening is spin relaxation. Hence, broadened ^31^P NMR lines observed in peptide monomers or fibrils should arise from inhomogeneous broadening owing to structural disorder and heterogeneities, as reflected in a broadened distribution of isotropic ^31^P NMR chemical shifts.

The ^31^P NMR spectra of Y310^p^ fibrils and monomers of both S305^p^ and Y310^p^ exhibit substantial line broadening (550–700 Hz), consistent with inhomogeneous broadening from structural heterogeneity and packing disorder within these samples. In contrast, the narrow ^31^P NMR spectral component of the S305^p^ fibril sample is only ∼210 Hz. This linewidth reduction observed at both low and high NaCl concentrations, but only in distinct spectral components of the S305^p^ fibrils (Figures 4 and S2), reflects greater structural homogeneity and minimized variations in isotropic ^31^P chemical shifts (distinct from CSA). Next, we investigate whether this greater order originates from association and unique spatial arrangements of phosphoryl groups within the S305^p^ fibril architecture.

### 2.5 MQ‐SC Experiments

To evaluate inter‐phosphoryl group proximity and ordering, we carry out Multiple‐Quantum Spin Counting (MQ‐SC) experiments. Our goal was to determine the minimal cluster size by analyzing the highest possible extracted MQ coherence order (MQCO), which is equal to the minimal number of coupled ^31^P spins present within a spin cluster. The structure of the phosphorylated jR2R3 P301L fibrils will dictate the spatial arrangement of ^31^P spins in S305^p^ or Y310^p^ residues within the β‐sheets. The different spin network geometries that MQ‐SC experiments are sensitive to include parallel and anti‐parallel arrangements, or trimer and protofibril arrangements with an internuclear distance of 4–5 Å [19]. In an in‐register and parallel packed β‐sheet structure, the ^31^P spins on adjacent tau fibrils should form a nearly linear arrangement with internuclear distances of ∼4.8 Å if the phosphoryl groups associate with each other across the β‐sheet. However, if the phosphoryl groups repel each other, the protein site harboring the phosphoryl group would be disordered, and no significant MQ‐CO would be observed.

**FIGURE 5:**
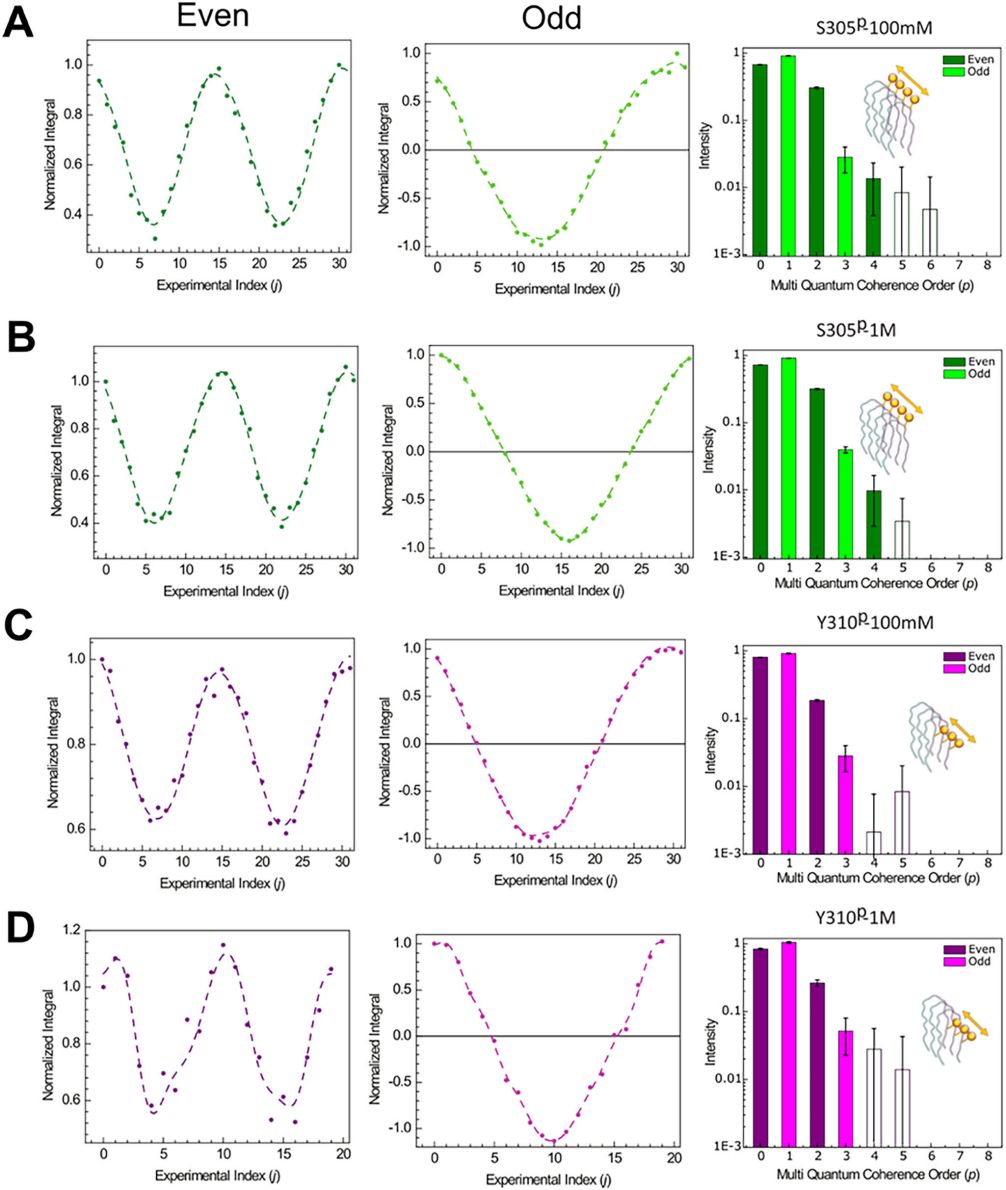
Experimental even and odd Multiple Quantum Spin Counting (MQ‐SC) profiles acquired at 10 kHz MAS frequency and with 8 ms excitation time using a SR2^1^_8_ DQ recoupling sequence with a repetition time of 5 s under DNP conditions. The signal integral is plotted as a function of the experimental index (j), where each phase is incremented by 360°/j, normalized to the integral of the first experiment (*j* = 0) (left, middle). Charts of the intensity for the Multiple Quantum Coherence Order (*p*) are obtained from multi‐cosine curve fitting of the MQ‐SC profiles of S305^p^ jR2R3 P301L fibrils and Y310^p^ jR2R3 P301L fibrils and plotted on a logarithmic scale as a function of *p* (right). The MQ‐SC profiles and MQCO charts are presented for (A) 100 mM NaCl S305^p^, (B) 1 M NaCl S305^p^, (C) 100 mM NaCl Y310^p^, and (D) 1 M NaCl Y310^p^.

MQ‐SC experiments were performed on S305^p^ fibrils (prepared in the presence of [NaCl] of 1 M or 100 mM) while varying the excitation times between 4.8 and 9.6 ms using the SR2^1^_8_ sequence (shown in Figure S11). Upon calibration, an excitation time of 8 ms was used for MQ‐SC experiments on all fibril and monomer samples in this study. The phase of the excitation block was incremented in phase steps of 2π/*j* (*j* = number of experiments) to extract the MQCOs for *p* = 0 to *j*/2, while all other pulse and detection phases in the experiment were kept constant. Figure 5 illustrates the MQ‐SC profiles of even and odd spin‐counts and the intensity chart of the corresponding MQCOs (*p* from 0 to 8) extracted for S305^p^ and Y310^p^, respectively, under both 100 mM and 1 M NaCl conditions. We utilized a multi‐cosine fitting method to extract the MQCOs from the MQ‐SC profiles, a new analysis method for counting MQCOs [40] that relies on the knowledge that the spin counting profiles are a superposition of multi‐cosine waveforms, as elucidated by the Average Hamiltonian Theory described by Oyler and Tycko [46]. The conventional Fourier transformation (FT) technique generates all frequencies, thereby producing all possible MQCOs. We showed that the FT analysis procedure is less robust for determining the lower bound frequencies in the MQCO profile, which corresponds to the highest observable MQCO [40]. The extracted MQCO intensities and the error values corresponding to each sample are listed in Table S7. Because MQ‐SC profiles will show fluctuations even in the absence of any MQCO, it is the intensity relative to the error bar that determines whether the MQCO count is real. The narrow component in the S305^p^ spectra is the dominant spectral component of the S305^p^ fibrils prepared at 100 mM and 1 M NaCl concentration and is found to have MQCOs up to *p* = 4 above error (indicated in filled green) for both preparations. In contrast, the two broader components of S305^p^ fibrils do not exhibit MQCO beyond 2. In contrast, the maximum MQCO observed for Y310^p^ fibrils for the total spectral component was 3. The MQ‐SC profiles of the monomeric S305^p^ and Y310^p^ peptides, not subjected to aggregation reactions, show random fluctuation (displayed in Figure S14) as a function of phase increment of the excitation block, and hence no meaningful MQCO could be identified. Although an MQCO of 3 in Y310^P^ fibrils is less than that of 4 in S305^P^ fibrils, the phosphoryl group stacking still has greater ordering in Y310^p^ fibrils than in vitrified peptide monomers.

To investigate the assembly structure of phosphoryl groups in S305^p^ fibrils that give rise to an MQCO of 4, a starting point for the spatial ^31^P arrangement is needed. According to high‐resolution cryogenic transmission electron microscopy (cryo‐EM) structures of tau fibrils from post‐mortem brain tissues of tauopathy patients, tau proteins assemble in register parallel stacking along the fibril axis (examples shown in Figure S16) [7, 42, 47]. The inter‐tau spacing in fibrils is ∼4.8 Å across all patient‐derived tau fibrils, while tau proteins in the disease context are hyperphosphorylated, and at some sites, at high abundance. It follows that a linear arrangement of inter‐phosphoryl arrangement is a good starting point from MD simulations to find the free‐energetically stable ^31^P arrangements on fibrils of S305^P^ that can be used to perform the MQ‐SC simulations; this will be discussed later. Qualitatively, we can conclude that an MQCO of 4 in S305^P^ fibrils implies neat and close phosphoryl group stacking.

The peptides were deliberately designed with a single phosphoryl group on a given tau peptide chain to avoid interference effects from intra‐molecularly proximal (5–7 Å spacings) phosphoryl groups in fibrils made of doubly phosphorylated tau. Effects of divalent cations (Mg^2^^+^ or Ca^2^^+^) were also excluded as Mg^2^^+^ associates strongly with phosphate and phosphoryl groups and can cause precipitation through enhanced inter‐protein associations. This design allows us to ask whether a single phosphoryl group on tau can drive inter‐molecular association into a 1D wire arrangement through inter‐phosphoryl attraction, independent of divalent cation or intra‐protein folding effects. The MQ‐SC results show that the phosphoryl groups closely associate, while the GdnHCl assay shows that phosphoryl groups reinforce supramolecular fibril assembly despite their net negative charge. We hypothesize that phosphoryl groups form extended “wires” through water‐mediated hydrogen‐bonding that stabilizes tau fibrils.

**FIGURE 6:**
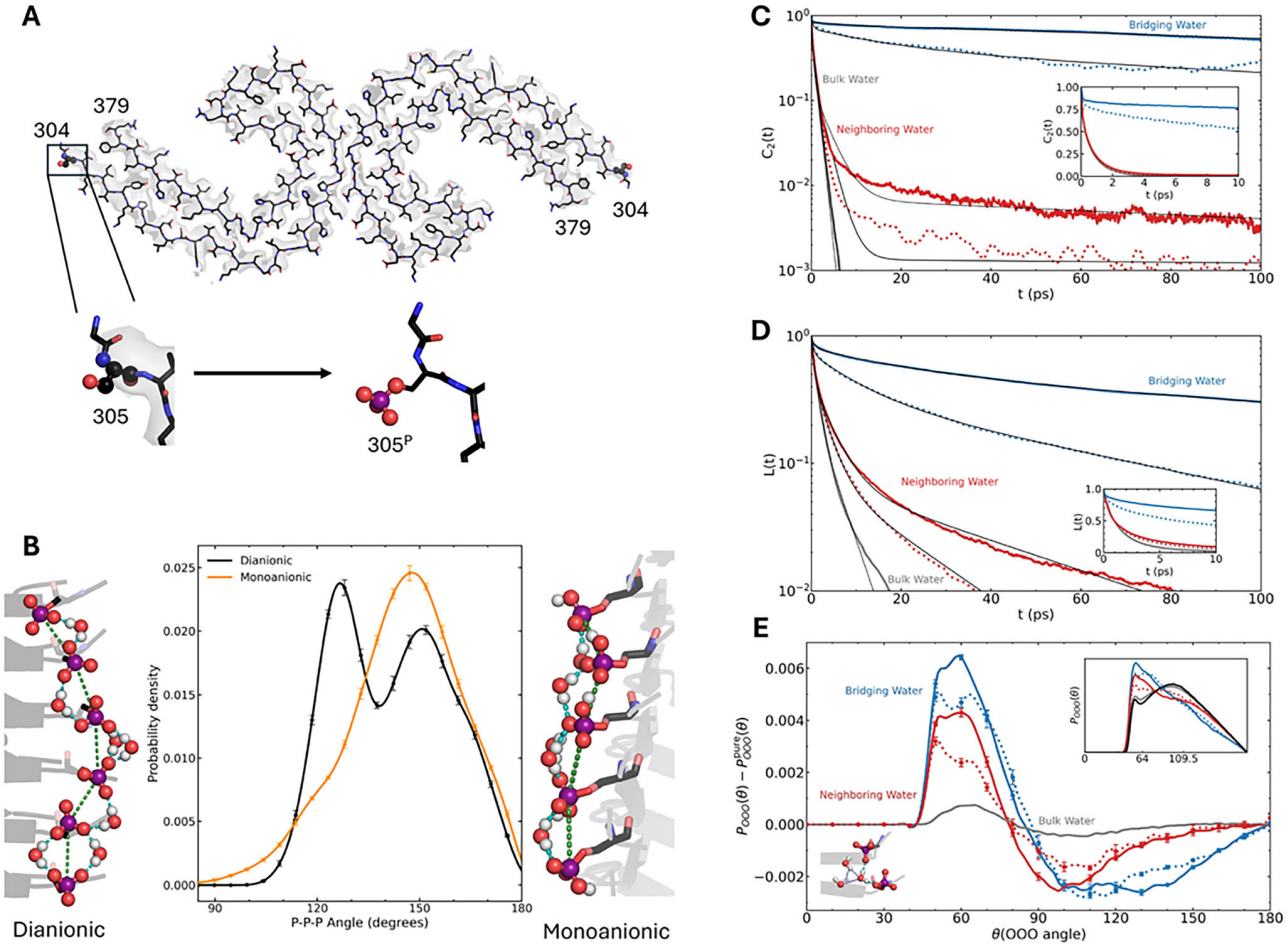
Visualization of structured water bridges formed near residues S305^p^ of an eight‐layer fibril (PDB ID #7QL4) [26] and comparative analysis of water dynamics from simulations. (A) Cryo‐EM density map of the in vitro paired helical filament‐like structure from Alzheimer's disease (PDB: 7QL4) [26] in the transparent gray superimposed with the atomic structure. The inset represents the non‐phosphorylated serine at residue 305 and a phosphorylated serine for one layer of the fibril. (B) Probability density of three‐body angle distribution of adjacent phosphorus atoms in the phosphoryl groups that form bridging waters with neighboring phosphoryl groups for the dianionic (black) and monoanionic (orange), omitting the terminal layers, shown alongside bridging waters in a representative structure. Cyan dashes represent the hydrogen bonds of the bridging waters with the terminal oxygens (red) of the phosphoryl groups. Green dashes represent the P–P–P angle formed by adjacent phosphorous atoms (purple). Error bars represent the standard deviation of five blocks from block averaging at select angles. (C) Rotational anisotropy autocorrelation function 𝐶_2_(𝑡) of the bridging waters (blue), neighboring waters (red), and bulk water (gray) for the dianionic (solid) and monoanionic (dashed) on a semi‐log scale with exponential fits (black). Bulk water was fitted with a biexponential function (τ = 0.58 ps). Neighboring waters were fitted with a triexponential function for the dianionic and monoanionic cases (see Section S2.0). (D) Hydrogen bond lifetime correlation function 𝐿(𝑡) of the various waters on a semi‐log scale with exponential fits (black) for the dianionic (solid) and monoanionic (dashed) phosphoryl groups. A biexponential fit was used for bulk water, and a triexponential fit was used for neighboring and bridging waters function for the dianionic and monoanionic cases (see Section S2.0). (E) Differential three‐body angle distributions. The inset plot shows the three‐body angle distributions of neighboring waters and bridging waters (line: dianionic, dash: monoanionic) with a peak consistent with icosahedral geometry (𝜃 ≈ 64°). Error bars show standard deviation for a subset of angles in bridging and neighboring waters from block averaging with 5 blocks. The three‐body angle distribution of bulk water shows a peak consistent with tetrahedral geometry (𝜃 ≈ 109.5°). The black curve represents the three‐body angle distribution of pure water at 310 K. A representative water bridge is shown depicting the three‐body angle (O–O–O).

Next, we turn our attention to the geometrical arrangement and energetics of phosphorylated tau fibrils using molecular dynamics (MD) simulations performed in explicit water. As a representative model structure for *β*‐sheet packing and spacing, we selected an in vitro paired helical filament‐like structure from Alzheimer's disease (PDB ID #7QL4) [26] (Figure 6A). The precise fibril fold is not critical here, since the goal is to determine whether water molecules can stabilize phosphoryl groups along the exterior surface of a tau fibril in the absence of structural constraints that would otherwise favor linear phosphoryl group assemblies. The fibril exterior serves solely as a scaffold, which is a feature common to all amyloid fibrils, with the ∼4.8 Å spacing between tau strands along the *β*‐sheet defining the approximate ^3^ ^1^P–^3^ ^1^P spacing, as discussed earlier.

To determine the viability and stability of phosphoryl‐wires, the physiologically relevant charge state of the phosphoryl groups must be considered. Serine at residue 305 was modeled as dianionic phosphoserine, given that a previous study reported the dianionic form to be the dominant species at pH = 7.5, the monoanionic form to dominate at pH = 3.6, and the neutral form at pH = 1.1 [48]. Consistent with this, the reported pKa of 5.6 for the monoanionic–dianionic equilibrium of phosphoserine predicts that the dianionic form predominates at the experimental condition of pH 7.4, while the monoanionic form remains non‐negligible at pH 6.0, both of which represent physiologically relevant conditions [49]. Accordingly, energy minimization was carried out for both the dianionic and monoanionic forms.

Energy minimization of the phosphorylated fibril structure, followed by NVT and NPT equilibration simulations, was carried out. A 100 ns trajectory was run, and the last 2 ns of the trajectory were analyzed to capture the water structure, dynamics, and bonding of waters with and surrounding the phosphoryl groups. The details of the MD simulation are provided in Section S1.9 of the Materials and Methods section. When screening through the frames in the trajectory of energetically favorable structures, water molecules have been found to form bridges between phosphoryl groups by establishing hydrogen bonds (hydrogen bond criterion is provided in Section S2.0) for both dianionic and monoanionic forms.

The three‐body P–P–P angle between the phosphorous atoms connected by continuous bridging waters across neighboring layers provides an effective measure of the linearity of the phosphoryl arrangement within the fibril (Figure 6B). Distributions of this angle were obtained for both the monoanionic and dianionic forms of the phosphorylated fibril. In both cases, the P‐P‐P angle distribution is relatively broad, spanning 110° to 180°, reflecting a dynamic, partially ordered phosphoryl wire. The monoanionic state exhibits a more linear P–P–P angle distribution (greater population at 150°) than the dianionic state (greater population at 120°), the latter resembling a wire with a zig‐zag geometry. In both cases, the fibrils are stabilized by water‐mediated hydrogen bonds between anionic phosphoryl groups.

**FIGURE 7:**
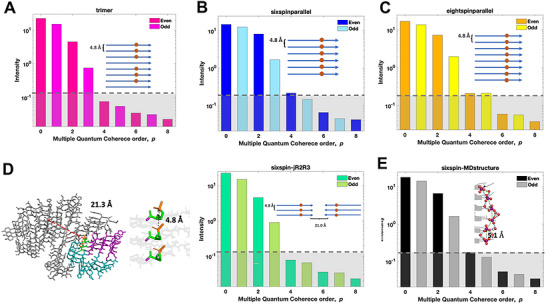
Model fibril of phosphorylated tau and numerical MQCO charts obtained by SIMPSON simulations for different geometries of a linear three, six, eight‐spin systems, protofibril triple‐stack geometry, and an MD‐derived six‐spin geometry. Numerical MQCO charts for a (A) trimer geometry, (B) six‐spin parallel configuration, (C) eight‐spin parallel configuration, (D) proto fibril geometry in a triple stack from the structure of jR2R3 fibrils [42], and (E) representative MD structure (shown in Figure 6B of dianionic state). (D) A tilted top view of the jR2R3 P301L (PDB ID #8V1N) [42] fibril structure with phosphorylation at site S305, showing a distance of 21.3 Å shown between the two phosphoryl groups of adjacent protofibrils, and a distance of 4.8 Å between the phosphoryl groups of vertically stacked β strands of S305^p^ jR2R3. A detailed description of the spin system of each configuration is given in Table S8. The black line represents the average error derived from multi‐cosine function fitting to the MQ‐SC profiles. Intensity values on the y‐axis of the MQCO chart are plotted on a logarithmic scale.

Next, MQ‐SC simulations using SIMPSON [50, 51, 52] were performed for the following geometric models: (i) linear chains of 3, 6, and 8 spins with a spacing of 4.8 Å (Figure 7A–C); and (ii) a paired triple‐stack representing a protofibril geometry, with an inter‐stack distance of 21 Å based on the closest inter‐tau distance at site 305 in the cryo‐EM structure of unphosphorylated jR2R3‐P301L fibrils (Figure 7D) [42]. Ongoing cryo‐EM structure studies of the phosphorylated structure suggest a similarly long ^31^P‐^31^P distance within protofibrils. Even and odd MQ‐SC simulations used parameters identical to those employed experimentally for all spin network geometries (Tables S8 and S9). Finally, we examined (iv) a representative dianionic phosphoryl group arrangement from the MD structure, which is shown in Figure 6B with the corresponding ^31^P atoms adopting a zig‐zag geometry and average ^31^P‐^31^P distance of 5.1 Å (Figures 6B and 7E).

Trimer simulations yielded MQCOs of 1–3 (Figure 7A); a 6‐spin linear arrangement gave MQCOs up to 4 (Figure 7B); and an 8‐spin linear arrangement gave MQCOs up to 5 (Figure 7C). The protofibril triple‐stack geometry with a ^3^ ^1^P–^3^ ^1^P inter‐protofibril distance of 21 Å yielded MQCOs up to 3 (Figure 7D), confirming that distant inter‐protofibril ^3^ ^1^P–^3^ ^1^P spacings do not contribute to the observed MQCOs (Table S5). Small variations in chemical shifts did not affect the MQCOs in the triple‐stack simulations, though larger variations would reduce them (Figure S13B). If the inter‐protofibril ^3^ ^1^P–^3^ ^1^P distance exceeds 5 Å, an MQCO of 4 implies parallel in‐register stacking of at least six tau molecules; if it is less than 5 Å, this effect would increase the apparent MQCO amplitude. Still, an MQCO of 4 requires at least three neatly packed phosphoryl groups. Note that high MQCOs are harder to observe in vitrified samples with shorter T_2_ relaxation times and weaker dipolar couplings (<180 Hz at 4.8 Å). The MD‐derived 6‐spin zigzag geometry also yielded MQCO 4 (Figure 7E), with slightly lower amplitude than the linear case, attributable to the longer inter‐phosphoryl distance (∼5.16 Å) and correspondingly weaker dipolar coupling; nonetheless, the near linear P–P–P angle makes this geometry functionally analogous to the 6‐spin linear arrangement. We conclude that the experimental MQ‐SC results are consistent with a 1D ^31^P wire arrangement of a stack of six phosphoryl groups.

Our MD simulations of phosphorylated tau fibrils in explicit water showed that bridging water molecules stabilize the phosphoryl groups and promote linear geometries, as discussed earlier. To quantitatively examine the contribution of hydration‐mediated interactions to this structural organization, we compared the populations of three distinct water species interacting with the phosphoryl groups: bulk water, neighboring water molecules within 5 Å of residue 305, and bridging waters hydrogen‐bonded to oxygens connecting two adjacent phosphoryl groups. Various parameters were calculated for all three types of water: *C*_2_(*t*) from the rotational anisotropy autocorrelation function (ACF) (Figure 6C) [53], hydrogen bond lifetime correlation function *L*(*t*) (Figure 6D) [54, 55], and the differential three‐body angle distributions $\;\Delta P_{3b}\left(\theta \right)=P_{3b}\left(\theta \right)-P_{3b}^{\mathrm{pure}}\left(\theta \right)$ (Figure 6E). Definitions of these quantities are provided in the Sec S2.0. The rotational anisotropy ACF provides insights into restrictions on structural dynamics of water in confined regions. Decay times and hydrogen bond lifetimes were extracted by fitting the ACFs and L(*t*) to exponential functions. Bulk water was fit with a biexponential for *C*_2_(t) and L(*t*), while bridging and neighboring waters required triexponential fits, yielding three distinct lifetimes corresponding to three populations. The corresponding fitting plots are shown in Figure 6C,D, with extracted values in the caption. Bridging waters for the dianionic case showed an order‐of‐magnitude longer orientational decay times, τ_2_ = 5.0 ps (5%), τ_3_ = 259.4 ps (79%), with τ_1_ = 0.10 ps for the remaining 16%—compared to neighboring waters (τ_2_ = 3.0 ps, 15%; τ_1_ = 0.39 ps, 84%) and bulk water (τ = 0.58 ps). Bridging waters also showed significantly longer hydrogen bond lifetimes (τ_1_ = 0.67 ps, τ_2_ = 21.5 ps, τ_3_ = 186.1 ps) relative to both bulk and neighboring waters. Similar trends are observed for the monoanionic phosphoryl groups, but the bridging and neighboring waters do not exhibit as slow of a decay compared to the hydration waters involved in the dianionic phosphoryl groups.

Water structuring also plays a crucial role in driving the interactions between water and the fibril surface [42]. The three‐body (O‐O‐O) angle distribution, *P*_3b_(θ) captures changes in water structure near the fibril surface [56, 57] and was calculated for all waters within a cutoff distance of 3.5 Å of a phosphoryl group. The differential three‐body angle distribution, Δ*P*_3b_(θ), for both monoanionic and dianionic phosphoryl groups, reveals that neighboring waters have a higher population in the tetrahedral range (100°< θ < 120°), slightly exceeding that of bridging waters. Bulk waters exhibited characteristic icosahedral (θ = 64°) populations along with a positive feature near θ = 50°, corresponding to over‐coordinated water molecules [58, 59]. Integrating over the tetrahedral region of *P*_3b_(θ) (100°< θ < 120°) gives percentages of water with tetrahedral geometries: pure water (310 K) 21.4%, bulk water 20.62%, neighboring waters 17.50% (18.51%), and bridging waters 16.61% (16.07%) for dianionic (monoanionic) phosphoryl groups. The higher tetrahedrality observed in neighboring waters relative to bridging waters suggests structured water formation around the phosphoryl groups, likely driven by local hydrophobic effects commonly referred to as “wrap waters.” [60] A similar trend is observed for both monoanionic and dianionic forms. Together, these MD results indicate that bridging water molecules are structurally constrained, exhibit significantly longer hydrogen bond lifetimes, and do not readily exchange with bulk water. The simulations further demonstrate that bridging water molecules mediate extended phosphoryl “wires” through hydrogen bonding, that these wires represent free, energetically stable configurations, and that bridging water may be a critical factor in stabilizing the fibril.

**FIGURE 8:**
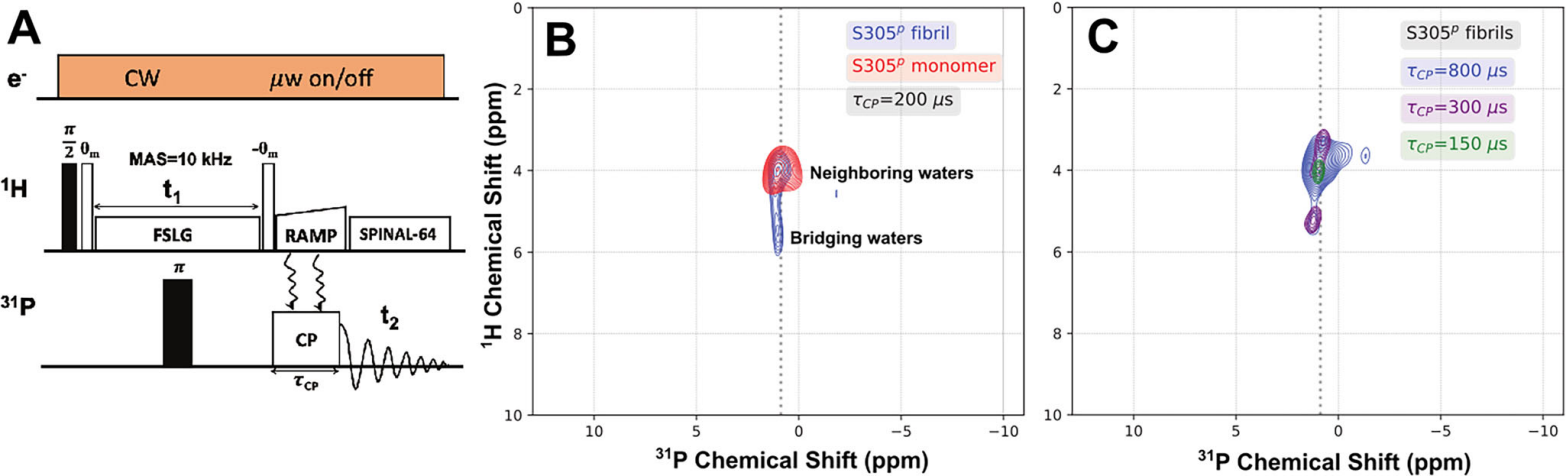
(A) Pulse sequence for ^1^H‐^31^P FSLG–HETCOR, and (B, C) 2D ^1^H‐^31^P FSLG–HETCOR data obtained at 10 kHz MAS frequency and 100 K temperature under DNP condition of (B) S305^p^ monomers (red) and S305^p^ fibrils (blue) with a CP contact time of 200 µs, and (C) S305^p^ fibrils with different CP contact times of 150, 300 and 800 µs. The fibrils were prepared in 100 mM NaCl. Further experimental details are given in Section S1.6.2.

To obtain experimental evidence for the water‐mediated phosphoryl group correlations identified by MD simulations, we performed 2D ^1^H‐^31^P HETCOR experiments on S305^p^ monomers and fibrils in 100 mM NaCl (Figure 8B,C). At a CP contact time (τ_CP_) of 200 µs, the spectra show a narrow ^3^ ^1^P NMR signal for fibrils and a broader signal for monomers, consistent with the ^3^ ^1^P 1D NMR lineshapes discussed earlier; the 1D ^1^H cross‐sections along the ^1^H dimension are shown in Figure S9. The broad ^3^ ^1^P component correlates with a ^1^H resonance at 4.0–4.5 ppm, while the narrow ^31^P component, observed only in the S305^p^ fibril sample, shows an additional correlation with a ^1^H NMR signal at ∼5.5 ppm. We assign the 4.0–4.5 ppm signal to bulk water, and the downfield‐shifted 5.5 ppm signal to hydrogen‐bonded water protons dynamically bridging two phosphoryl‐group oxygens, where the sandwiched protons are rendered more acidic by exchange with the phosphoryl P‐OH groups. This assignment is supported by literature reports that water protons directly associated with phosphoric acid and phosphoryl groups are downfield‐shifted relative to bulk water due to chemical exchange among H_3_PO_4_, H_2_O, and H_3_O^+^, with shifts reported as high as 8.6 ppm [61] and the expected shift of phosphoryl‐bound water protons near 5–6 ppm. The 4.0–4.5 ppm correlation along the ^1^H dimension is observed at all contact times tested down to τ_CP_ = 150 µs, whereas the 5.5 ppm feature requires τ_CP_ = 200 µs or longer, indicating that the narrow ^3^ ^1^P component is either more distant from, or less coupled to, nearby protons than the broader spectral component.

The ^1^H(4 ppm)‐^31^P(1 ppm) cross‐peak in the 2D spectrum (Figure 8C) broadens along the ^31^P spectral dimension with CP contact time, indicating that both the narrow and broad ^31^P components of the S305^p^ fibril are in proximity to bulk water. In contrast, the ^1^H peak at 5.5 ppm shows no broadening along the ^31^P dimension, even for long CP contact times up to *τ*_CP_ = 800 µs. This result indicates that the water molecules giving rise to this resonance interact exclusively with the narrow ^31^P spectral component of S305^P^ fibrils, corresponding to phosphoryl groups arranged in an extended 1D wire, and do not interact with heterogeneous ^31^P species outside this arrangement, nor do they exchange with bulk water protons. Taken together, the chemical shift value and the HETCOR cross‐peak features allow this resonance to be assigned to a discrete population of water molecules bridging adjacent phosphoryl groups. The selective correlation with the narrow ^31^P component and the absence of bulk water exchange are in excellent agreement with the MD predictions of constrained, long‐lived bridging water molecules that stabilize the extended phosphoryl wire arrangement.

To evaluate whether phosphorylation stabilizes fibrils by promoting aggregation‐prone conformations rather than inter‐phosphoryl group assembly, we examined fibril structure and monomer conformational dynamics. MD simulations show no structural deformation of phosphorylated relative to unphosphorylated R2R3 tau fibrils, with canonical β‐sheet spacing preserved at 4.7–4.8 Å (Figure S16), ruling out steric repulsion or structural distortion as the driving mechanism. ^1^H‐^15^N HSQC spectra (Figure S17) of monomeric jR2R3 with and without phosphorylation show comparable linewidths and negligible ^1^H and ^15^N chemical shift differences, indicating that phosphorylation does not pre‐organize aggregation‐prone monomer conformations or promote oligomerization. It should be noted that carboxylate groups used in pseudophosphorylation or sulfate groups are not appropriate mimics for studying inter‐phosphoryl group association, given their differences in geometry, hydrogen bonding capacity, and charge distribution relative to phosphoryl groups. While sulfated heparin polymers can align along the surface of amyloid fibrils, this reflects electrostatic association driven by a polymer with substantial persistence length, rather than the water‐mediated network assembly observed specifically with phosphoryl groups. Collectively, these results support a water‐mediated phosphoryl‐wire stabilization mechanism rather than steric or conformation‐based models.

The MQ‐SC results reinforce the hypothesis that the phosphoryl groups in S305^p^ fibrils form a stabilizing 1D wire, giving rise to the dramatic ^3^ ^1^P line narrowing observed by NMR. The phosphoryl groups in Y310^p^ fibrils associate favorably and enhance fibril stability, but do not form an extended 1D arrangement. Together, these findings indicate that phosphoryl group association, rather than strict 1D ordering, is the primary mechanism of fibril stabilization. To our knowledge, MQ‐SC is the only method capable of providing direct insight into both the number of phosphoryl groups participating in ordered extended structures and their spatial arrangement, information that cannot be obtained from conventional spectroscopic methods.

## 3 Conclusion

The molecular‐level mechanisms by which phosphorylation influences intermolecular protein interactions and tau aggregation have remained poorly understood. Fibril denaturation experiments using guanidinium hydrochloride (GdnHCl) revealed that phosphorylated jR2R3 fibrils are significantly more stable than their unphosphorylated counterparts, with S305^p^ fibrils exhibiting the greatest stability, an effect we attribute to higher‐order association between phosphoryl groups. 1D ^31^P NMR spectra and MQCO experiments further demonstrate that phosphorylation of serine at site S305 dramatically enhances both phosphoryl group ordering and fibril stability. MD simulations corroborated this mechanism, revealing thermodynamically stable, water‐mediated, phosphoryl wire geometries, and 2D ^1^H–^3^ ^1^P HETCOR spectra provided direct experimental evidence that these 1D phosphoryl wires are stabilized by water‐mediated hydrogen bonding between phosphoryl groups on adjacent tau molecules. Together, these results identify a previously unrecognized role of phosphorylation in promoting tau fibril packing and stability through tight, in‐register alignment of phosphoryl groups into an extended 1D arrangement, with implications for the misfolding pathway in pathological tau aggregation. Given that hyperphosphorylation is among the most prevalent PTMs and a key biomarker of pathological tau proteins under neurodegenerative disease conditions and the basis of the AT8 antibody used to identify AD pathology, which recognizes phospho‐S202 and phospho‐T205 [62], we propose that ordered phosphoryl wires may constitute a relevant structural hallmark of pathological tau fibrils. As fibrils with greater ordering and stability are known to exhibit greater seeding competency, we further propose that phosphoryl wire formation in S305^p^ tau fibrils may confer enhanced pathological potency through this mechanism.

Even in this 19‐residue peptide with only two potential phosphorylation sites, the effects of phosphorylation are highly site‐specific and context‐dependent, determined in part by whether the modified site lies within a stable tau fibril core. The propensity of phosphate and phosphoryl groups to self‐associate has been largely overlooked, in part because the major role of bound water in delocalizing charge across multiple water molecules has only recently been recognized. The net negative charge of phosphate/phosphoryl groups, regardless of protonation state, is distributed such that the hydrogen bond‐forming propensity of the coordinated hydration water molecules is reinforced by the phosphate/phosphoryl groups. The critical role of water in these associations represents a regime distinct from the conditions that favor covalent ^31^P─^31^P bond formation in a polymerization process. The tetrahedral hydrogen‐bonding geometry of water molecules hydrating the phosphoryl groups permits a wide range of long‐range spatial arrangements, unconstrained in all dimensions except along the axis defined by the phosphoryl‐tau attachment. This extended organization of water‐mediated phosphoryl group assembly was identified by ^31^P MQCO experiments that rely on generating higher‐order (MQ) coherences among correlated, dipolar‐coupled ^31^P spins. Further investigation of the structural and dynamic properties of these phosphoryl wire assemblies, including the effect of divalent cations such as Mg^2^^+^ or Ca^2+^ on their structure and/or stability, will be the subject of future work. MQ‐SC studies of fibrils formed from different tau proteoforms are expected to shed light on the aggregation pathways in tau pathologies.

## Conflicts of Interest

The authors declare no conflicts of interest.

## Supporting information



Fibril preparation, details of stability studies, NMR experimental details, MQSC simulation details, MD simulation details, functions used in MD analysis for rotational anisotropy, hydrogen bond lifetime, overlay of ^31^P NMR spectra of S305^p^ and Y310^p^ with their monomers, decoupling effect on the ^31^P spectra, ^31^P NMR spectra simulated using SIMPSON with CSA, dipolar couplings with different MAS, Fourier transformed MQCO profiles of S305^p^ and Y310^p^, MQCOs extracted for monomers, TEM images from fibril denaturation experiment, 2D ^1^H‐^15^N HSQC spectra, parameters obtained from line shape fitting of the 1D ^31^P spectra, CSA parameters obtained of all the samples using the CSA‐MAS model in DMfit, description of the spin systems used for 1D SIMPSON simulations, MQCO intensities and the error values of all samples obtained by multi‐cosine wave function fitting, description of spin‐systems used for MQSC, SR2^1^_8_ DQ‐SQ SIMPSON script.
**Supporting File**: anie72568‐sup‐0001‐SuppMat.pdf.

## Data Availability

The data that support the findings of this study are available from the corresponding author upon reasonable request.
