# Supplementary material for: Water‐Mediated Phosphoryl Wires Stabilize Pathological Tau Fibrils
Source: Angew Chem Int Ed Engl. 2026 May 23;65(30):e21499. doi: 10.1002/anie.202521499 (PMC13383178; doi:10.1002/anie.202521499)
Supplement: Supplementary file 1 — Fibril preparation, details of stability studies, NMR experimental details, MQSC simulation details, MD simulation details, functions used in MD analysis for rotational anisotropy, hydrogen bond lifetime, overlay of 31P NMR spectra of S305p and Y310p with their monomers, decoupling effect on the 31P spectra, 31P NMR spectra simulated using SIMPSON with CSA, dipolar couplings with different MAS, Fourier transformed MQCO profiles of S305p and Y310p, MQCOs extracted for monomers, TEM images from fibril denaturation experiment, 2D 1H‐15N HSQC spectra, parameters obtained from line shape fitting of the 1D 31P spectra, CSA parameters obtained of all the samples using the CSA‐MAS model in DMfit, description of the spin systems used for 1D SIMPSON simulations, MQCO intensities and the error values of all samples obtained by multi‐cosine wave function fitting, description of spin‐systems used for MQSC, SR21 8 DQ‐SQ SIMPSON script. Supporting File: anie72568‐sup‐0001‐SuppMat.pdf. [file ANIE-65-e21499-s001.pdf]

Supporting Information of
Water-Mediated Phosphoryl Wires Stabilize Pathological Tau Fibrils

Lokeswara Rao Potnuru[a], Austin DuBose[b], Fiona Mon[c], Mesopotamia S. Nowotarski[b], Michael Vigers[b], Boqin Zhang[b], Chung-Ta Han[a], John E. Straub[c], Songi Han[a,b,d*]

[a] Lokeswara Rao Potnuru, Chung-Ta Han, Songi Han

Department of Chemistry, Northwestern University, Evanston 60208 Illinois, United States of America

E-mail: songi.han@northwestern.edu

[b] Austin DuBose, Mesopotamia S. Nowotarski, Michael Vigers, Boqin Zhang, Songi Han

Department of Chemistry and Biochemistry, University of California Santa Barbara, 93106, California, United States of America

[c] Fiona Mon, John E. Straub

Department of Chemistry, Boston University, Boston, 02215, Massachusetts, United States of America

[d] Songi Han

Department of Chemical Engineering, University of California Santa Barbara, 93106, California, United States of America

Contents:

Fibril preparation and stability studies

NMR experimental details, MQSC simulations

Molecular dynamics details, and the functions used for its analysis

Overlay of ^{31}P NMR spectra of S305^{p} and Y310^{p} with their monomers, decoupling effect on the ^{31}P spectra

Simulations for 1D line shape analysis using SIMPSON with CSA, dipolar couplings with different MAS

Fourier transformed MQCO profiles of S305^{p} and Y310^{p}

MQCOs extracted for monomers

TEM images from fiber denaturation experiment

2D ^{1}H-^{15}N HSQC spectra of jR2R3-P301L peptide monomers of (non)phosphorylated

Parameters from line shape fitting of the ^{31}P spectra, CSA parameters obtained of the different samples

Description of the spin systems used for 1D SIMPSON simulations

MQCO intensities and the error values of all samples obtained by multi-cosine wave function fitting

Description of the spin systems used for SIMPSON simulations, SR2^{1}_{8} DQ-SQ SIMPSON script

1. Materials and Methods

1.1 Fibril Preparation Method

All jR2R3 P301L peptides were purchased from GenScript and powders were dissolved to 1 mM stock solutions and stored at -80° C or used immediately. The aggregation process was performed in a 50 mL Eppendorf tube. The stock peptide was diluted to 50 μM using 20 mM ammonium acetate, pH 7.4 (calibrated with HCl and NaOH), with either 100 mM or 1 M NaCl and accompanied by a 4:1 (peptide to heparin) molar ratio of additional heparin (average molecular weight 16 kDa). Continuous shaking was performed at 200 rpm at 37° C for 24 hours. Fibrils were collected into a 50 kDa cutoff concentrator and rinsed 3 times with MilliQ water to remove monomeric peptide and excess heparin. Next, fibrils were transferred to a weighed Eppendorf tube and lyophilized using a FreeZone 2.5 Liter -84° C Benchtop Freeze Dryer overnight. The mass of each fibril sample was quantified.

1.2 Thioflavin T Aggregation Assay

All experiments were performed with a BioTek Synergy 2 fluorescent plate reader. In each well, jR2R3 P301L (50 μM), ThT (20 μM), and heparin (12.5 μM) in buffer were distributed into a 384-well plate (Corning low volume non-binding surface black with clear flat bottom) to a total volume of 30 μL. The plate reader temperature was set to 37 °C and allowed to equilibrate, the samples were shaken under the programmed high shaking speed in between measurements. ThT fluorescence intensity was measured at (excitation=440 nm, emission=485 nm) every 2 minutes until a plateau was reached. These experiments were done in triplicate at least three times with independent samples.

1.3 TEM Analysis

For transmission electron microscopy (TEM) analysis, five μL of fibril samples were applied to a glow-discharged copper grid (Electron Microscopy Science, FCF-200-Cu) for 20 s and blotted dry with filter paper. Samples were stained with 5 μL 1.5 w/v % uranyl acetate and immediately blotted dry. An additional 5 μL of uranyl acetate was added for 60 seconds and blotted dry. Samples were analyzed using a Thermo Scientific Talos G2 200X TEM/STEM microscope operated at 200 kV and room temperature. Grids were then imaged with a Ceta II CMOS 4k x 4k camera at the indicated magnifications.

1.4 Fibril Stability by Denaturation

Guanidinium Hydrochloride denaturation assays were performed using identical conditions to the initial fibrilization ThT assays. Prior to the stability tests and to ensure accurate ThT assessment during stability testing, it was necessary to remove any excess monomers and heparin. This was achieved by washing the fibrils in their original buffer containing 20 mM ammonium acetate and 100 mM NaCl using a concentrator with a 50 kDa cutoff. To achieve the specified GdnHCl concentration for the reported results, the fibrils' initial concentration was reduced through dilution into the denaturation buffer. The values reported were obtained following 24 hours after the signal reached equilibration upon initial incubation.

1.5 In Vitro Seeded Aggregation Protocols

Fibrils used for the seeding experiments were assembled using identical conditions to the initial fibrilization assay. Samples were reconstituted to a final concentration of 1 mM in the reaction buffer. The seeds were subjected to sonication for 30 seconds using the microtip of a Qsonica sonicator, operating at a 30% duty cycle. Seeding assays were performed in a Tecan Spark, replicates were pipetted in a 384 well plate clear with black bottom, at a final concentration of 50 μM and a total volume of 30 μL. Seeds were added in a 1 monomer: 1 seed ratio.

1.6 Solid-State NMR Experimental Details

The lyophilized powdered fibrils were dissolved into DNP juice, prepared with 6:3:1 parts of glycerol-d8, D_2O, and H_2O with 10 mM AMUPol (Cortecnet, C010P005). The above samples with a final concentration of 4 mM (0.5 mg of fibril in 40 μl of DNP juice) were centrifuged into regular

walled sapphire 3.2 mm MAS rotors and plugged with Teflon or silicon inserts to prevent leakage and subsequently injected into the spectrometer at 100 K. All the MAS DNP measurements were carried out on a Bruker Avance III DNP-NMR 400 MHz spectrometer equipped with a 25 W gyrotron microwave source (operating at 263 GHz frequency) at a temperature of 100 K. A triple resonance H/X/Y probe was used in double resonance mode with a 50 Ohm resistor on the Y channel. The signal enhancement of 40-fold was obtained using the DNP method with samples in the sub-milligrams (0.4 to 0.5 mg) regime. One-dimensional ^{31}P spectra were collected by using ^{1}H-^{31}P cross-polarization experiments. MQ-SC experimental data was obtained using the SR2^1_8 pulse sequence for DQ excitation and reconversion. 2D ^{1}H-^{31}P HETCOR experiments were carried out to get the ^{1}H-^{31}P correlations. Further experimental details were given in the SI.

1.6.1 1D ^{31}P CP spectra

A pulse length of 2.8 μs was used for the excitation of the protons. For ^{31}P, a pulse of length 3.4 μs was used for 90º rotation. ^{1}H-^{31}P cross-polarization experiments were performed with a ramp on the ^{1}H channel by varying its rf amplitude from 70 % to 100 % and 94 kHz rf power on ^{1}H and 80 kHz rf power on the ^{31}P channel, respectively with sufficient contact time. During the ^{31}P acquisition, heteronuclear decoupling of 90 kHz rf power was employed on the ^{1}H channel. One-dimensional ^{1}H-^{31}P CP spectra were obtained by 128 transients for each sample with a recycle delay of 5 seconds. CP-echo spectra were obtained using CP followed by a spin-echo to measure homogeneous linewidth of the samples.

1.6.2 2D ^{1}H-^{31}P HETCOR spectra

Pulse lengths and the power levels similar to the 1D ^{1}H-^{31}P CP were used for acquiring the 2D ^{1}H-^{31}P HETCOR spectra. Frequency Switched Lee-Goldburg (FSLG) method was used to suppress the ^{1}H-1H homonuclear couplings and the subsequent transfer of magnetization from protons to phosphorous was done by ^{1}H-^{31}P cross-polarization (CP) with various contact times to observe the short range and long range ^{1}H-^{31}P correlations. 2D spectra were obtained by collecting 160 FIDs, each with 40 co-added transients using the States-TPPI method for indirect acquisition. A recycle delay of 5 seconds was used.

1.6.3 MQ-SC spectra

MQ-SC experiments were carried out using the SR2^1_8 pulse sequence for DQ excitation and reconversion.[1] SR2^1_8 works with DQ operators, and the spin counting experiments would generate an even number of multi-quantum coherences. The same SR2^1_8 sequence was used to get the odd number of multiple-quantum coherences by shifting the starting coherence to ±1 with a 90º pulse applied before and after the DQ excitation and reconversion, respectively. During the ^{31}P acquisition and recoupling duration, heteronuclear decoupling of 90 kHz rf power was employed on the ^{1}H channel. MQ-SC experiments were carried out with 256 scans. A relaxation delay of 5 seconds was used.

1.7 Data Processing

All NMR integrals taken for spin counting analysis were processed using a custom Python Jupyter Notebook code. Left shift, line broadening, baseline correction, and custom integrals were applied for all FIDs of a given data set similar to the procedure in our previous article.[1] MQCO profiles were very susceptible to phasing and left shift accuracy. Each FID was analyzed separately to ensure data was cohesive.

1.8 Quantum mechanical simulations of SSNMR experiments

Multiple-quantum SSNMR simulations were carried out using the SIMPSON package with a magnetic field strength of 9.4 T and MAS frequency of 10 kHz. SR2^1_8 pulse sequence was used for excitation and the reconversion blocks with a radiofrequency amplitude of 5 kHz, equal to half the spinning frequency which is the requirement for fulfilling the basic structure of the R2^1_8 symmetry sequence. 8 ms time was used for the excitation and reconversion blocks. Powder averaging was performed with a crystal file rep20 and ten gamma angles. Simulations for six and eight ^{31}P spin networks with different geometries were performed to explain and support the experimental results. The spin system was generated using the MagresView[2] web interface for

different geometries. Isotropic chemical shifts, CSA tensors, scalar couplings and heteronuclear couplings were not considered in the simulations since their magnitudes were smaller compared to the MAS frequency of 10 kHz and their contribution to the 1D ^{31}P spectra is negligible. The phase of the excitation block was incremented with a step of π/16 while keeping all other phases constant. The MQCOs were extracted by applying a Fourier Transform to the intensity profile obtained from the simulation.

1.9 MD Simulations

The *in vitro* cryo-EM tau fibril structure (PDB: 7QL4) was extended to eight layers respecting the fibril pitch. Serine at residue 305 was converted to dianionic and monoanionic phosphoserine and both the N- and C-terminal ends of each peptide were neutralized through the CHARMM-GUI PDB Reader and Manipulator.[3] The system was solvated with TIP3P model waters and 100 mM NaCl salt and simulated using GROMACS 2021.5 employing the CHARMM36m force field at a temperature of 310.15 K. [4,5] A rhombic dodecahedral box was used with a minimum distance of 2 nm from the fibril to the box edge. Energy minimization was performed with the steepest descent algorithm. A 5 ns NVT equilibration was performed with position-restraining harmonic forces applied to the backbone (400 kJ mol^{-1} nm^{-2}) and sidechains (40 kJ mol^{-1} nm^{-2}) using the velocity-rescaling thermostat.[6] Subsequently, a 5 ns NPT equilibration was performed with the same restraints using the velocity-rescaling thermostat and Berendsen barostat.[6,7] A third NPT equilibration was performed for 5 ns without restraints using the Nosé-Hoover thermostat and Parrinello-Rahman barostat.[8–10] A trajectory was run for 100 ns with a 2 fs timestep. Frames were saved every 0.1 ps for the last 2 ns of the simulation. Analysis of the system was performed on the last 2 ns of the trajectory with MDAnalysis.[11]

2.0 Supplement to molecular dynamics simulation

To assess the linearity of the phosphoryl groups, the three-body P-P-P angle was calculated for only those phosphorous atoms forming continuous bridging waters in neighboring layers, omitting the terminal layers. Three different water groups were defined: bulk water, neighboring waters within 5 Å of residue 305, and bridging waters that were hydrogen bonded to the oxygens between two adjacent phosphoryl groups. Bridging waters were identified using a hydrogen bond donor-acceptor distance cutoff of 3.5 Å and a donor-hydrogen-acceptor angle cutoff of 120°. The rotational anisotropy correlation function was defined:

$$C_2(t) = \langle P_2(\hat{u}(t) \cdot \hat{u}(0)) \rangle$$

where $P_2(x) = (3x^2 - 1)/2$ is the second Legendre polynomial and $x = cos\,\theta = \hat{u}(t) \cdot \hat{u}(0)$, where $\hat{u}$ is the unit vector of the O-H bonds.[12]

The hydrogen bond lifetime correlation function was defined:

$$L(t) = \left\langle \frac{h_{ij}(t+\tau)h_{ij}(\tau)}{h_{ij}(\tau)^2} \right\rangle$$

where $h_{ij}(\tau) = 1$ indicates that there is a hydrogen bond between atoms *i* and *j* at time τ and $h_{ij}(t+\tau) = 1$ indicates that atoms *i* and *j* remain hydrogen bonded throughout the period τ to $t + \tau$, ignoring bond breaking within five frames (0.5 ps).[13,14] For the water bridges, the hydrogen bond lifetime correlation function was computed by tracking whether one hydrogen of a water molecule remained hydrogen bonded to the oxygen of a phosphoryl group on one chain, while simultaneously the other hydrogen remained bonded to the oxygen of a phosphoryl group on an adjacent chain. The rotational anisotropy and hydrogen bond lifetime correlation functions were computed using multiple time origins, with the result obtained by averaging all origins to enhance statistical accuracy. For the hydrogen bond lifetime of bulk water, a biexponential function was fitted:

$$L(t) = A_1 e^{\left(\frac{-t}{\tau_1}\right)} + (1 - A_1) e^{\left(\frac{-t}{\tau_2}\right)}$$

to obtain the amplitude-weighted effective hydrogen bond lifetime:

$$\tau = A_1\tau_1 + (1 - A_1)\tau_2$$

where τ is the hydrogen bond lifetime. From the biexponential fit for bulk water, $\tau_1 = 1.01$ ps, $\tau_2 = 4.17$, and $A_1 = 0.73$.

Rotational anisotropy autocorrelation function $C_2(t)$ was fit to a triexponential function for dianionic phosphoryl groups (τ_1=0.39 ps, τ_2=3.0 ps, τ_3=185.6 ps with weights A_1=0.84, A_2=0.15, and A_3=0.01) and monoanionic phosphoryl groups (τ_1=0.32 ps, τ_2=2.0 ps, τ_3=1000. ps with weights A_1=0.768, A_2=0.231, A_3=0.001). Bridging waters were fitted with a triexponential function for dianionic (τ_1=0.10 ps, τ_2=5.0 ps, τ_3=259.4 ps with weights A_1=0.16, A_2=0.05, and A_3=0.79) and monoanionic (τ_1=0.41 ps, τ_2=17.1 ps, τ_3=156.4 ps with weights A_1=0.29, A_2=0.30, and A_3=0.41).

Hydrogen bond lifetime correlation function $L(t)$ was fit to a triexponential function for neighboring water in the dianionic case (τ_1=0.57 ps, τ_2=4.01 ps, τ_3=36.8 ps with weights A_1=0.51, A_2=0.42, and A_3=0.07) and monoanionic case (τ_1=0.51 ps, τ_2=2.84 ps, τ_3=17.8 ps with weights A_1=0.43, A_2=0.48, and A_3=0.1). Bridging waters fit well to a triexponential function for the dianionic case (τ_1=0.67 ps, τ_2=21.5 ps, τ_3=186.1 ps with weights A_1=0.20, A_2=0.29, and A_3=0.51) and monoanionic case (τ_1=0.52 ps, τ_2=10.3 ps, τ_3=59.5 ps with weights A_1=0.27, A_2=0.39, and A_3=0.34).

The three-body (O-O-O) angle distribution for each water group was calculated by identifying water molecules adjacent to a central water molecule and within a cutoff distance of 3.5 Å. Angles were computed based on the positions of three oxygen atoms, consisting of the central water and two adjacent waters.

3. Supplement to experimental NMR data

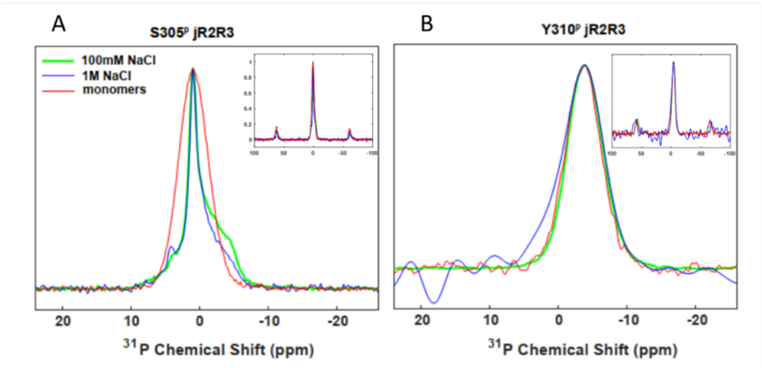

Fig. S1. Overlay of ^{31}P NMR spectra of A) S305ᵖ jR2R3 P301L and B) Y310ᵖ jR2R3 P301L samples, respectively.

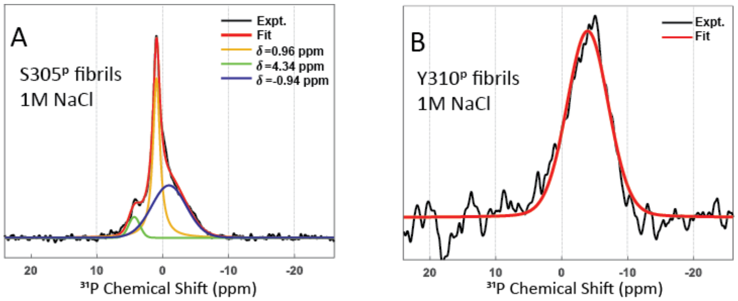

Fig. S2. ^{31}P NMR spectra of 1M NaCl fibrils of A) S305ᵖ jR2R3 P301L and B) Y310ᵖ jR2R3 P301L and the corresponding deconvolution of the spectra using DMfit software.

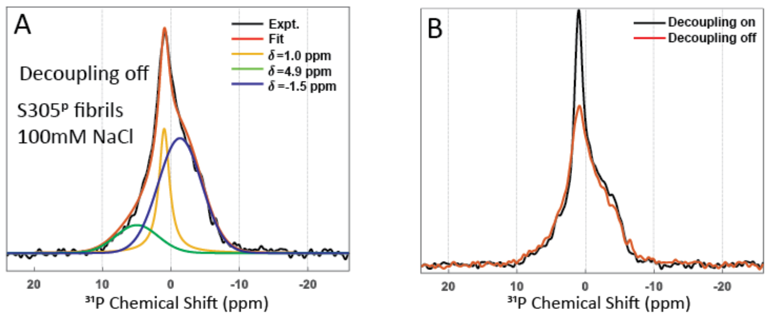

Fig. S3. The effect of ^{1}H-^{31}P heteronuclear decoupling on 100 mM NaCl S305^{p} ^{31}P spectra. A) Deconvolution of decoupled spectra of S305^{p} fibrils, B) ^{31}P spectra of S305^{p} fibrils with decoupling power on (black) / power off (red) on proton channel.

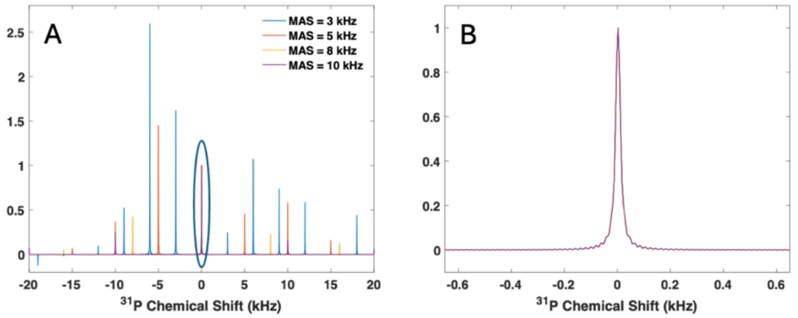

Fig. S4. A) The simulated ^{31}P spectra for a single ^{31}P spin at various MAS frequencies with a CSA of 100 ppm at 400 MHz field spectrometer. B) Zoomed version of the peak circled in A. An internal line broadening of 20 Hz was used.

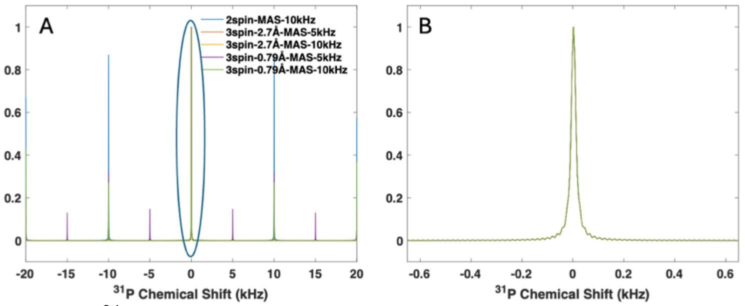

Fig. S5. A) The simulated ^{31}P spectra at MAS frequencies considering only dipolar couplings at 400 MHz field spectrometer. B) Zoomed version of the peak circled in A. An internal line broadening of 20 Hz was used.

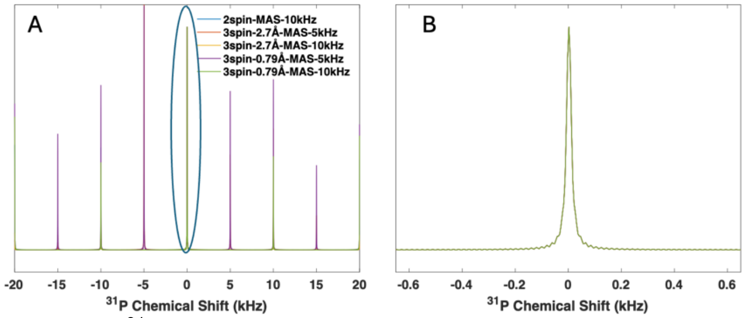

Fig. S6. A) The simulated ^{31}P spectra at MAS frequencies with a CSA of 100 ppm and dipolar couplings taken together at 400 MHz field spectrometer. B) Zoomed version of the peak circled in A. An internal line broadening of 20 Hz was used.

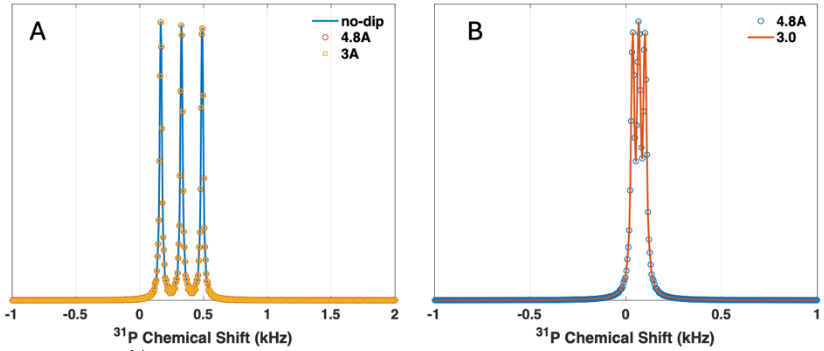

Fig. S7. The simulated ^{31}P spectra at MAS frequency of 10 kHz with a CSA of 100 ppm and dipolar couplings taken together at 400 MHz field spectrometer. A) Three spins with chemical shifts of 1, 2 and 3 ppm, B) Three spins with chemical shifts of 0.2, 0.4 and 0.6 ppm. An internal line broadening of 20 Hz was used. Spinning side bands were not shown.

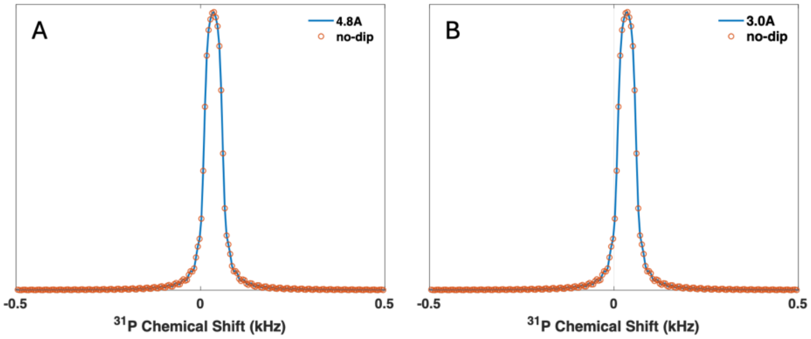

Fig. S8. The simulated ^{31}P spectra at MAS frequency of 10 kHz with a CSA of 100 ppm and with and without dipolar couplings taken together at 400 MHz field spectrometer. Three spins with chemical shifts of 0.1, 0.2 and 0.3 ppm were considered. A) simulations for 4.8Å, B) for 3Å distance between adjacent ^{31}P spins. An internal line broadening of 20 Hz was used. Spinning side bands were not shown.

3.1 Line broadening mechanisms in solid-state NMR

It is important to identify the factors responsible for linewidths in solid-state NMR spectra. Line broadening arises due to both homogeneous and inhomogeneous contributions. Homogeneous broadening originates due to coherent and incoherent interactions. The coherent contribution comes from the spin system Hamiltonian (CSA, homo and hetero nuclear dipolar couplings for spin ½). The incoherent contribution is due to stochastic processes i.e., relaxation and chemical exchange. On the other hand, inhomogeneous broadening arises from B_0 field inhomogeneities, sample heterogeneity, structural disorder due to the presence of multiple conformations, which give rise to distributions of isotropic chemical shifts.

So, the total line width in NMR can be given by the following equation

$$\Delta_{tot} = \Delta_{homo} + \Delta_{inhomo} = \Delta_{coh.} + \Delta_{incoh.} + \Delta_{inhomo}$$

The homogeneous linewidth can be obtained by measuring spin-relaxation time constant (T_2). CP-echo (cross-polarization followed by a Hahn-echo) pulse sequence was used to collect the data for all the samples. In S305^p, the spectra were deconvoluted to get the narrow and broad component signals. T_2 values were determined by fitting the normalized data to a single exponential $e^{-\frac{t}{T_2}}$ with T_2 as a fitting parameter. For the rest of the samples, the T_2 values were obtained using both single-exponential $e^{-\frac{t}{T_2}}$ and bi-exponential function ($ae^{-\frac{t}{T_2^a}} + be^{-\frac{t}{T_2^b}}$) and were listed in Table S4 and the corresponding T_2 derived homogeneous line widths were calculated by $\frac{1}{(\pi T_2)}$ and reported in Table S4. The inhomogeneous broadening for rest of the samples, based on the major population from the biexponential fit, was determined by subtracting the homogeneous linewidth from the total observed linewidth, with the values reported in Table S5. The inhomogeneous line widths were found in the range of 550-700 Hz across all broad signals, except for narrow component of S305^p. For this narrow component of S305^p, inhomogeneous broadening was found to be negligible, and its entire line width could be attributed to homogeneous broadening.

3.2 Contribution of homogeneous interactions to line width of ^{31}P spectra

Homogeneous broadening of the linewidth arises from both coherent and incoherent contributions. As discussed above, coherent homogeneous broadening originates from anisotropic interactions within the spin Hamiltonian, such as chemical shift anisotropy (CSA) and homo- and heteronuclear dipolar couplings. To first assess the effect of CSA, fits were performed using the CSA-MAS model in DMfit [15] software (fit values shown in Table S3). The CSA and asymmetry parameter (η) values were found to be similar between S305^p monomers and fibrils, as well as between Y310^p fibrils and monomers. The CSA was found to be on the order of 80-100 ppm across all the samples, corresponding to 13-16 kHz at the ^{31}P Larmor frequency of 162.0026519 MHz. Under MAS, CSA will only manifest in the intensities of the spinning side bands, not in the line width of center band or the side bands, indicating that CSA alone does not account for the observed ^{31}P NMR line width.

To understand the line broadening arising due to homogeneous broadening of CSA and ^{31}P-^{31}P dipolar couplings, we carried out the numerical simulations using SIMPSON at different MAS frequencies with only CSA (shown in Figure S4), only dipolar couplings (shown in Figure S5) and CSA and ^{31}P-^{31}P dipolar couplings taken together (shown in Figs. S6, S7 and S8). We did simulations for two cases, one having identical chemical shifts and second set of simulations with different isotropic chemical shifts.

First, we studied the system with identical chemical shifts with various conditions. The spectra shown in Figure S4, obtained for a single ^{31}P spin with a CSA of 100 ppm at various MAS frequencies, showed no increase in linewidth but did display changes in the separation of the spinning sidebands with varying MAS frequencies. Next, we examined the role of ^{31}P-^{31}P dipolar couplings in simulations (shown in Figure S5) at 10 kHz MAS on a three-spin system. Both weaker couplings (178 Hz between adjacent phosphorus spins corresponding to 4.8Å for inter-beta strands) and stronger couplings (730 Hz for 3Å distance) were tested, yet no linewidth increase was observed, because these couplings were an order of magnitude smaller and readily averaged out to first order under MAS. Even if we set the MAS frequency to 5 kHz in simulations, it averaged

out the ^{31}P-^{31}P dipolar couplings and orientation dependence of the dipolar vectors and producing a single narrow peak. Also, the higher order terms (second order terms of 4-5 Hz) also will be averaged out due to their smaller magnitudes under MAS. All these indicate that homonuclear ^{31}P-^{31}P dipolar couplings alone do not contribute to the linewidth. And then, we verified the contribution of CSA and homonuclear dipolar couplings taken together through the simulations at 10 kHz MAS frequency in Figure S6, we did not find any increase in the line width due to CSA x ^{31}P-^{31}P dipolar coupling cross-terms indicating that 10 kHz MAS frequency is sufficient to average out the CSA x ^{31}P-^{31}P dipolar terms in the spin system Hamiltonian.

In Figs. S7 and S8, we showed the simulation results on a three-spin system carried under MAS of 10 kHz having three sets of chemical shifts of 1, 2, and 3 ppm (Figure. S7A), 0.2, 0.4, and 0.6 ppm (Figure. S7B), and 0.1, 0.2, 0.3 ppm in the dipolar coupling regime of 4.8Å (corresponds to 178 Hz) (Figure. S8A) and 3Å (corresponds to 730 Hz) (Figure. S8B) between the adjacent ^{31}P spins. Under this dipolar coupling regime, the three chemical shifts behave as separate entities in the spectra Figs. S7A and S7B. In Figure S8A, B, it seems like a broadening, however this was verified with and without dipolar couplings, both look identical indicating that there was no line width contribution arising from cross-terms between CSA and ^{31}P-^{31}P dipolar couplings under 10 kHz MAS in the dipolar coupling regime of 4.8Å and 3Å. Here it appears like broadening because the isotropic chemical shifts separation of 16.2 Hz is less than line broadening of 20 Hz applied. So, the dispersion arising from the distribution of isotropic chemical shifts will add to the line broadening of the spectra.

The next anisotropic interactions to consider for coherent linewidth contributions are heteronuclear dipolar couplings, including ^{1}H-^{31}P, ^{13}C-^{31}P, and ^{15}N-^{31}P. Applying heteronuclear decoupling on protons during acquisition would eliminate or reduce the effects of ^{1}H-^{31}P couplings. We verified the effect of heteronuclear decoupling on ^{31}P spectra of 100 mM NaCl S305^{p} using the decoupling power on and power off (shown in Figure S3, line width values were given in Table S1) on the proton channel during the acquisition. We deconvoluted the ^{31}P spectra obtained by decoupling off condition and shown in Figure S3A. Indeed, line-narrowing upon proton decoupling was observed across all components in the spectra of S305^{p} fibrils, with a minimal reduction of 80 Hz in the narrow component (1.0 ppm peak) and a more pronounced reduction of 420 Hz in a broad component (4.9 ppm peak). These results showed that residual ^{1}H-^{31}P couplings were further reduced across all samples by applying decoupling on ^{1}H channel (Figs.4 and S3B). The contribution from ^{13}C-^{31}P and ^{15}N-^{31}P couplings is negligible due to their smaller magnitudes at natural isotope abundance for ^{13}C and ^{15}N. Thus, heteronuclear dipolar couplings are not the primary source of line broadening. Overall, we conclude that homogeneous coherent interactions arising from anisotropic terms (CSA, homo- and heteronuclear dipolar couplings) do not significantly contribute to the linewidths of the ^{31}P spectra. Instead, homogeneous broadening originates mainly from incoherent processes (stochastic relaxation mechanisms).

Therefore, based on the above discussions, the reduction in linewidth in the ^{31}P spectra of S305^{p} fibrils, observed at both NaCl conditions (Figs. 4 and S2A), may originate from enhanced structural homogeneity in neatly and stably packed fibrils that display minimal variation in the isotropic ^{31}P chemical shifts (different from CSA). In contrast, the phosphoryl groups of Y310^{p} fibrils and monomers of both S305^{p} and Y310^{p} exhibit 550-700 Hz of inhomogeneous broadening arising due to significant structural heterogeneity and packing disorder within the fibrils which result in the distribution of isotropic chemical shifts.

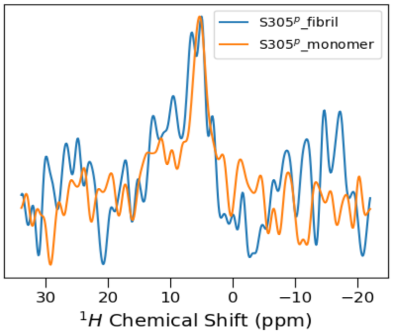

Fig. S9. The 1D slices extracted along ^{1}H dimension through the 2D ^{1}H-^{31}P spectra obtained with a CP contact time of 200 μs of 100 mM NaCl S305^p fibril and its monomer sample.

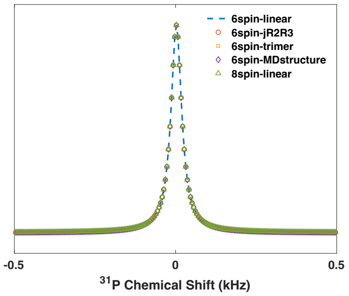

Fig. S10. The simulated ^{31}P spectra at MAS frequency of 10 kHz for different geometries used in MQSC simulations at 400 MHz field spectrometer and the corresponding spin systems are described in Table S8.

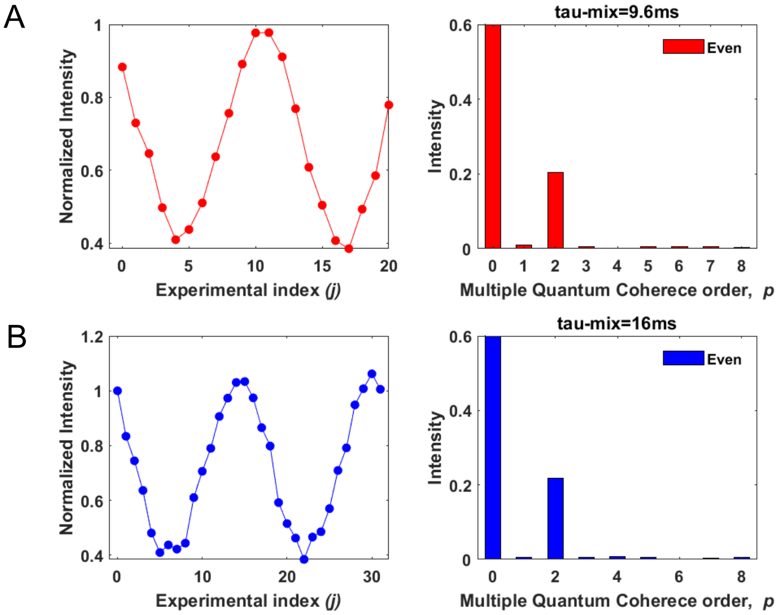

Fig. S11. Even order spin counting experiments with different SR2$^1_8$ mixing times of A) 9.6 ms and B) 16 ms on the ^{31}P spectra of 1M NaCl S305^p jR2R3 P301L fibrils.

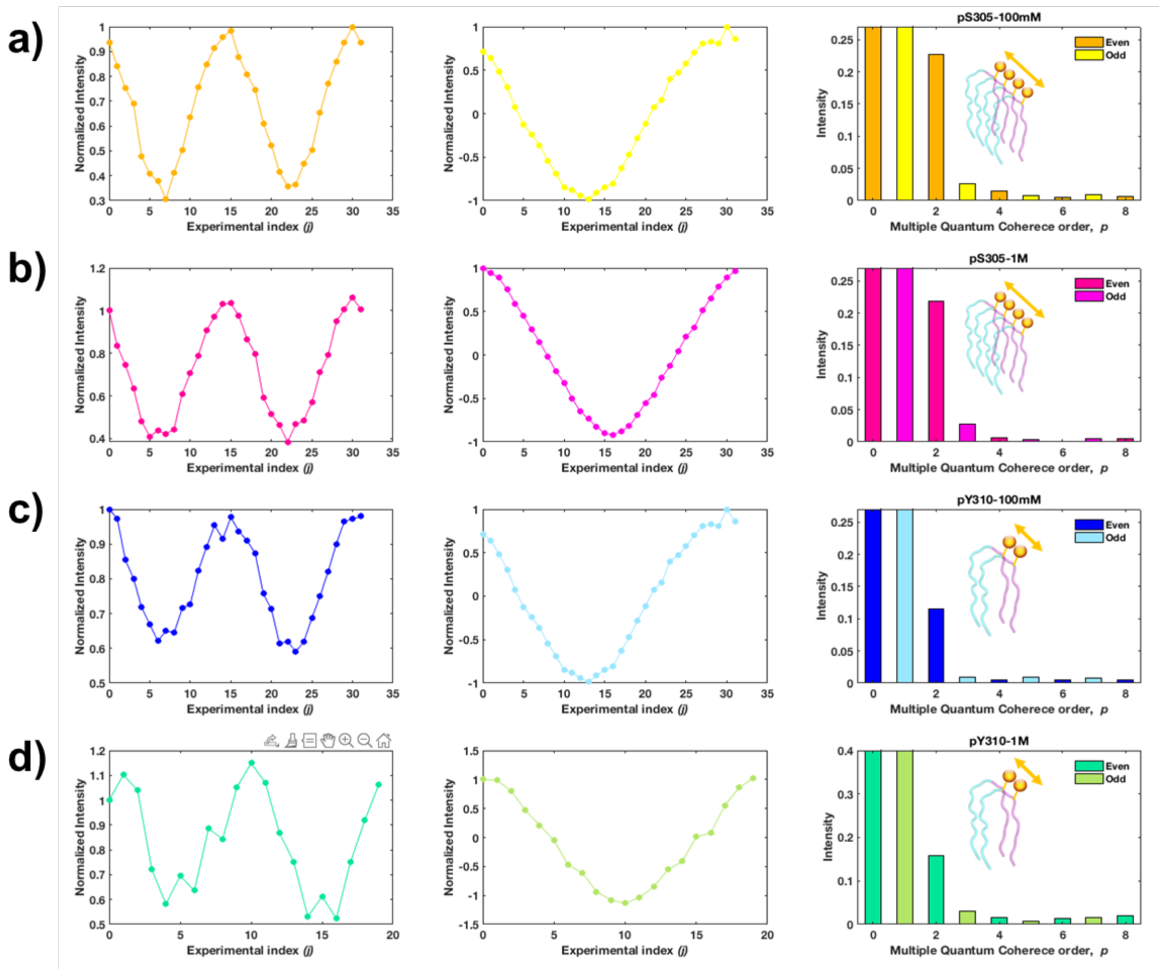

Fig. S12. Even and odd spin counting profiles and subsequent Fourier transformed MQCO profiles of S305^p jR2R3 fibrils and Y310^p jR2R3 fibrils at 10 kHz MAS frequency and with 8 ms excitation time during $SR2^1_8$ DQ recoupling sequence under DNP. (a) 100 mM NaCl S305^p, (b) 1 M NaCl S305^p, (c) 100 mM NaCl Y310^p, and (d) 1 M NaCl Y310^p.

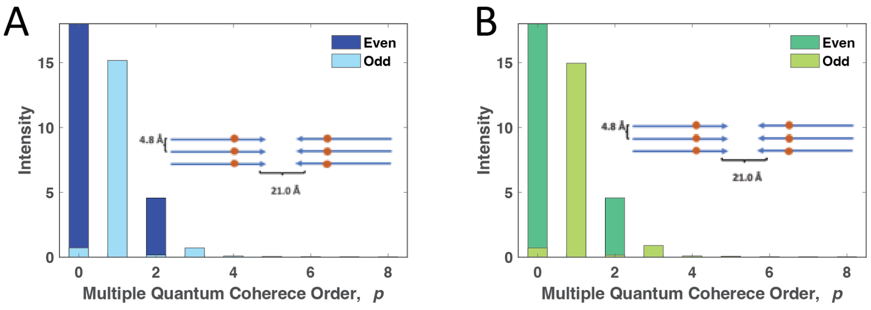

Fig. S13. Numerical SIMPSON simulations of MQ coherence orders of a six-spin system: A) triple stack of protofibril geometry from the structure of jR2R3 with 21 Å distance between the core and the counter strand [16], B) same geometry of A with small variations in the chemical shifts. Description of the spin system of each configuration is given in Tables S8 and S9.

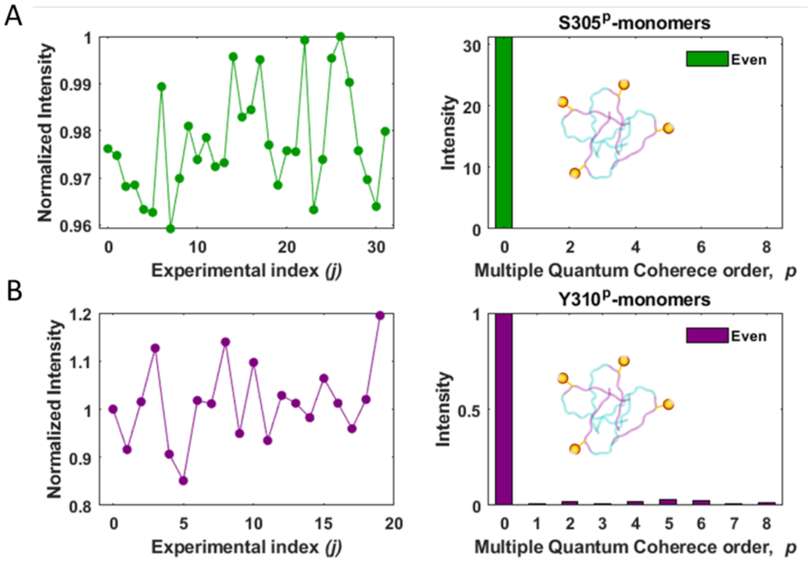

Fig. S14. Even MQ-SC profiles and MQCOs extracted from conventional FT method for monomers of S305ᵖ and Y310ᵖ jR2R3 P301L at 10 kHz MAS rate and 100 K temperature under DNP. (A) S305ᵖ jR2R3 P301L monomers and (B) Y310ᵖ jR2R3 P301L monomers. The x-axis of the spin counting profiles is represented by the experimental index (j), where each phase is incremented by 360°/experimental index. All spin-counting profiles' integrals were normalized to the integral of the first experiment (j = 0).

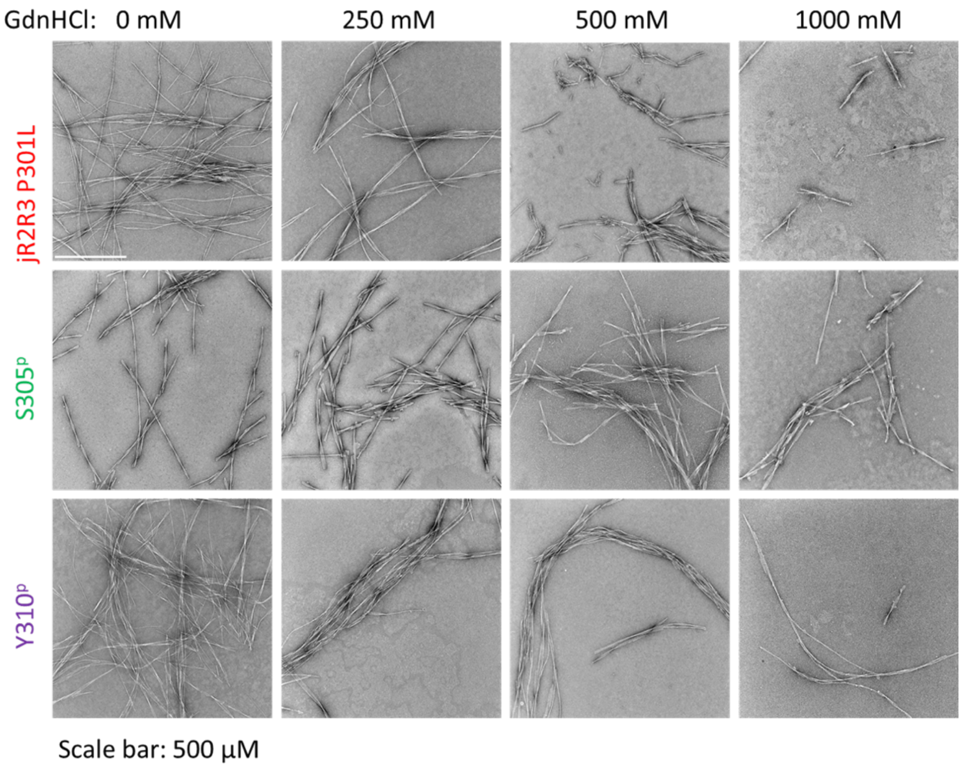

Fig. S15. TEM images from fiber denaturation experiment on fibrils of jR2R3 P301L (top), 100 mM NaCl S305ᵖ (middle) and 100 mM Y310ᵖ (bottom) fibril samples.

A

β-Sheet Stack Spacing by Tauopathy

Monomer-to-monomer spacing (cross-β rise) reported or inferred from cryo-EM structures of disease-derived tau fibrils

Tauopathy	Dominant Tau Isoform	Protofilament Rise (Å/monomer)
Alzheimer's disease (AD)	3R + 4R	~4.7 Å
Chronic traumatic encephalopathy (CTE)	3R + 4R	~4.7 Å
Pick's disease (PiD)	3R only	~4.7–4.8 Å
Corticobasal degeneration (CBD)	4R	~4.7 Å
Progressive supranuclear palsy (PSP)	4R	~4.7 Å
Argyrophilic grain disease (AGD)	4R	~4.7 Å (estimated)
Globular glial tauopathy (GGT)	4R	~4.7 Å (estimated)

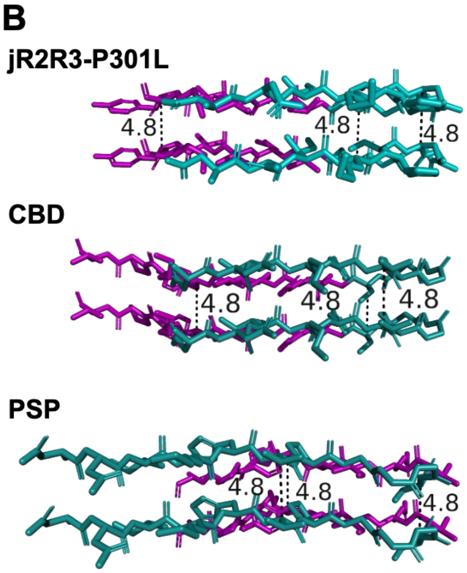

Fig. S16. A) Beta-sheet spacing for known Tauopathies, B) Top: jR2R3-P301L (PDB: 8V1N[17]), Middle: CBD (PDB: 6VH7[18]), and Bottom: PSP (PDB 7P65[19]). Only residues 295-313 shown for clarity. Spacing was checked at Site: 301, 305 and 310, all demonstrating a consistent 4.8 Å distance.

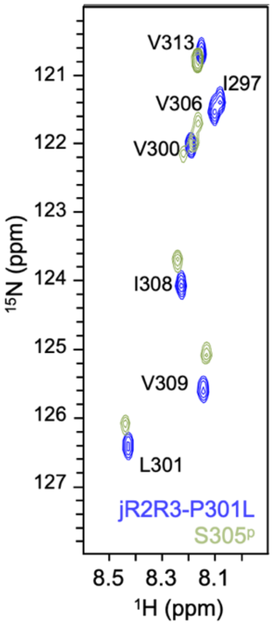

Fig. S17. Overlay of 2D ^{1}H-^{15}N HSQC spectra of jR2R3-P301L peptide monomers with (blue) and without phosphorylation (olive green).

Table S1. Line shape fitting of the ^{31}P spectra by Gaussian/Lorentzian (G/L) using DMfit. Values in the parenthesis were obtained from non-decoupled spectrum of Figure S3A.

Sample type	δ_{iso} (ppm)	FWHM (Hz)	Area (%)
100mM_NaCl_S305^{p} (narrow)	1.01	210 (290)	41
100mM_NaCl_S305^{p} (broad)	-1.55	1043 (1200)	51
100mM_NaCl_S305^{p} (broad)	4.85	750 (1170)	8
1M_NaCl_S305^{p} (narrow)	0.96	206	45
1M_NaCl_S305^{p} (broad)	-0.94	1008	49
1M_NaCl_S305^{p} (broad)	4.34	330	6
Monomers_S305^{p}	0.95	820	100
100mM_NaCl_Y310^{p}	-3.86	980	100
1M_NaCl_Y310^{p}	-3.92	1200	100
Monomers_Y310^{p}	-3.81	995	100

Table S2. Line shape fitting of the ^{31}P spectra by Gaussian/Lorentzian (G/L) using DMfit.

Sample type	1.0 ppm		4.85 ppm		-1.55 ppm	
**S305^{p}**	**G**	**L**	**G**	**L**	**G**	**L**
100mM_NaCl	0.0	1.0	1.0	0.0	1.0	0.0
1M_NaCl	0.0	1.0	1.0	0.0	0.95	0.05
Monomers	0.92	0.08	--	--	--	--
100mM_NaCl_nodec	0.0	1.0	1.0	0.0	1.0	0.0
**Y310^{p}**	**-3.9 ppm**					
100mM_NaCl	0.85	0.15				
1M_NaCl	0.9	0.1				
Monomers	0.9	0.1				

Table S3. T_2 measurements across all the samples.

Sample	Mono-exponential		Bi-exponential (a*exp(-t/T_2^a) + b*exp(-t/T_2^b))					
	T_2 (ms)	LW (Hz)	a	T_2 (ms)	LW (Hz)	b=1-a	T_2 (ms)	LW (Hz)
S305^p (narrow)	1.5	212	-	-	-	-	-	-
S305^p (broad)	0.77	410	-	-	-	-	-	-
S305^p monomer	1.29	250	0.87	1.18	270	0.13	9.6	30
Y310^p fibril	1.11	286	0.77	0.87	365	0.23	5.8	55
Y310^p monomer	1.27	250	0.80	0.93	315	0.20	4.5	65

Table S4. Line width estimation for the major population in all samples.

Sample	Line width (Hz)		
	Total (Hz)	Homo (Hz)	Inhomo (Hz)
S305^p (narrow)	211	212	0
S305^p (broad)	1040	410	630
S305^p monomer	800	250	550
Y310^p fibril	986	286	700
Y310^p monomer	960	250	690

Table S5. CSA parameters obtained for all samples by using the CSA-MAS model in DMfit.

**Sample type**	**δ_{iso} (ppm)**	**CSA (ppm)**	**Asymmetry parameter (η)**	**FWHM (Hz)**
100mM_NaCl_S305^p (narrow)	0.99	-75.0	0.7	200
100mM_NaCl_S305^p (broad)	-1.67	-81.0	0.78	1000
100mM_NaCl_S305^p (broad)	4.41	106	0.87	460
1M_NaCl_S305^p	0.94	-67.0	0.8	290
Monomers_S305^p	0.95	-85.0	0.75	800
100mM_NaCl_Y310^p	-3.86	-91	0.66	980
1M_NaCl_Y310^p	-3.92	-73	0.5	1000
Monomers_Y310^p	-3.81	-81	0.95	960

Table S6. Description of the spin systems used for SIMPSON simulations in Figure S4, S5, S6, S7 and S8.

Spin-system						
2-spin system	δ_{iso}	δ_{aniso}	η^{CS}	α_{PC}	β_{PC}	γ_{PC}
shift 1	0	100p	0	0	0	0
shift 2	0	100p	0	0	0	0
	$b_{ij}/(2\pi)$	α_{PC}	β_{PC}	γ_{PC}		
dipolar 1 2	-20000	0	0	0		
3-spin system (4.8A)	δ_{iso}	δ_{aniso}	η^{CS}	α_{PC}	β_{PC}	γ_{PC}
shift 1	0	100p	0	0	0	0
shift 2	0	100p	0	0	0	0
shift 3	0	100p	0	0	0	0
	$b_{ij}/(2\pi)$	α_{PC}	β_{PC}	γ_{PC}		
dipolar 1 2	-180	0	0	0		
dipolar 1 3	-22	0	0	0		
dipolar 2 3	-180	0	0	0		
3-spin system (3.0A)	δ_{iso}	δ_{aniso}	η^{CS}	α_{PC}	β_{PC}	γ_{PC}
shift 1	0	100p	0	0	0	0
shift 2	0	100p	0	0	0	0
shift 3	0	100p	0	0	0	0
	$b_{ij}/(2\pi)$	α_{PC}	β_{PC}	γ_{PC}		
dipolar 1 2	-730	0	0	0		
dipolar 1 3	-80	0	0	0		
dipolar 2 3	-730	0	0	0		
3-spin system (2.7A)	δ_{iso}	δ_{aniso}	η^{CS}	α_{PC}	β_{PC}	γ_{PC}
shift 1	0	100p	0	0	0	0
shift 2	0	100p	0	0	0	0
shift 3	0	100p	0	0	0	0
	$b_{ij}/(2\pi)$	α_{PC}	β_{PC}	γ_{PC}		
dipolar 1 2	-1000	0	0	0		
dipolar 1 3	-400	0	0	0		
dipolar 2 3	-1000	0	0	0		

3-spin system (0.79A)	δ_{iso}	δ_{aniso}	η^{CS}	α_{PC}	β_{PC}	γ_{PC}
shift 1	0	100p	0	0	0	0
shift 2	0	100p	0	0	0	0
shift 3	0	100p	0	0	0	0
	$b_{ij}/(2\pi)$	α_{PC}	β_{PC}	γ_{PC}		
dipolar 1 2	-40000	0	0	0		
dipolar 1 3	-4000	0	0	0		
dipolar 2 3	-40000	0	0	0		

Table S7. MQCO intensities and the error values shown in Figure 5.

MQCO	100 mM S305^{p}		1M S305^{p}		100 mM Y310^{p}		1M Y310^{p}	
	Amplitude	Error	Amplitude	Error	Amplitude	Error	Amplitude	Error
0	0.675	0.007	0.73	0.0048	0.8012	0.0039	0.839	0.02
1	0.91	0.011	0.91	0.004	0.913	0.0117	1.049	0.029
2	0.304	0.009	0.316	0.0067	0.185	0.0056	0.264	0.028
3	0.0282	0.011	0.395	0.004	0.028	0.0117	0.051	0.029
4	0.013	0.009	0.0096	0.0067	0.002	0.0056	0.027	0.029
5	0.008	0.011	0.0034	0.004	0.0084	0.0117	0.014	0.029
6	0.004	0.009	0.0001	0.0067	0.0007	0.0056	0.0234	0.029

Table S8. Description of the spin systems used for SIMPSON simulations in Figure 7 and S10.

Dipolar	$b_{ij}/(2\pi)$	α_{PC}	β_{PC}	γ_{PC}
Trimer				
dipolar 1 2	-178.2	-180.0	90.0	0.0
dipolar 1 3	-22.3	-180.0	90.0	0.0
dipolar 2 3	-178.2	-180.0	90.0	0.0
dipolar 4 5	-178.2	-180.0	90.0	0.0
dipolar 4 6	-22.3	-180.0	90.0	0.0
dipolar 5 6	-178.2	-180.0	90.0	0.0
Six-spin linear				
dipolar 1 2	-178.2	179.6	90.0	0.0
dipolar 1 3	-22.3	179.6	90.0	0.0
dipolar 1 4	-6.4	179.6	90.0	0.0
dipolar 1 5	-3.2	179.6	90.0	0.0
dipolar 1 6	-1.6	179.6	90.0	0.0
dipolar 2 3	-178.2	179.6	90.0	0.0
dipolar 2 4	-22.3	179.6	90.0	0.0
dipolar 2 5	-6.4	179.6	90.0	0.0
dipolar 2 6	-3.2	179.6	90.0	0.0
dipolar 3 4	-178.2	179.6	90.0	0.0
dipolar 3 5	-22.3	179.6	90.0	0.0
dipolar 3 6	-6.4	179.6	90.0	0.0
dipolar 4 5	-178.2	179.6	90.0	0.0
dipolar 4 6	-22.3	179.6	90.0	0.0
dipolar 5 6	-178.2	179.6	90.0	0.0
Eight-spin linear				
dipolar 1 2	-178.2	179.6	90.0	0.0
dipolar 1 3	-22.3	179.6	90.0	0.0
dipolar 1 4	-6.4	179.6	90.0	0.0
dipolar 1 5	-3.2	179.6	90.0	0.0
dipolar 1 6	-1.6	179.6	90.0	0.0
dipolar 1 7	-178.2	0.42	90.0	0.0

dipolar 1 8	-1.59	179.6	90.0	0.0
dipolar 2 3	-178.2	179.6	90.0	0.0
dipolar 2 4	-22.3	179.6	90.0	0.0
dipolar 2 5	-6.4	179.6	90.0	0.0
dipolar 2 6	-3.2	179.6	90.0	0.0
dipolar 2 7	-22.3	0.42	90.0	0.0
dipolar 2 8	-1.59	179.6	90.0	0.0
dipolar 3 4	-178.2	179.6	90.0	0.0
dipolar 3 5	-22.3	179.6	90.0	0.0
dipolar 3 6	-6.4	179.6	90.0	0.0
dipolar 3 7	-6.4	0.42	90.0	0.0
dipolar 3 8	-3.2	179.6	90.0	0.0
dipolar 4 5	-178.2	179.6	90.0	0.0
dipolar 4 6	-22.3	179.6	90.0	0.0
dipolar 4 7	-3.2	0.42	90.0	0.0
dipolar 4 8	-6.4	179.6	90.0	0.0
dipolar 5 6	-178.2	179.6	90.0	0.0
dipolar 5 7	-1.59	0.42	90.0	0.0
dipolar 5 8	-22.3	179.6	90.0	0.0
dipolar 6 7	-1.59	0.42	90.0	0.0
dipolar 6 8	-178.2	179.6	90.0	0.0
dipolar 7 8	0.0	179.6	90.0	0.0
Six-spin jR2R3-21Å				
dipolar 1 2	-178.2	90.0	90.0	0.0
dipolar 1 3	-22.3	90.0	90.0	0.0
dipolar 1 4	-1.6	25.6	90.0	0.0
dipolar 1 5	-1.6	13.5	90.0	0.0
dipolar 1 6	-3.2	0.0	90.0	0.0
dipolar 2 3	-178.2	90.0	90.0	0.0
dipolar 2 4	-1.6	13.5	90.0	0.0
dipolar 2 5	-3.2	0.0	90.0	0.0
dipolar 2 6	-1.6	-13.5	90.0	0.0
dipolar 3 4	-3.2	0.0	90.0	0.0
dipolar 3 5	-1.6	-13.5	90.0	0.0

dipolar 3 6	-1.6	-25.6	90.0	0.0
dipolar 4 5	-178.2	-90.0	90.0	0.0
dipolar 4 6	-22.3	-90.0	90.0	0.0
dipolar 5 6	-178.2	-90.0	90.0	0.0
Six-spin MD structure				
dipolar 1 2	-140.05	-177.6	90.0	0.0
dipolar 1 3	-19.1	169.1	90.0	0.0
dipolar 1 4	-6.36	170.6	90.0	0.0
dipolar 1 5	-3.18	-178.6	90.0	0.0
dipolar 1 6	-1.6	-165.4	90.0	0.0
dipolar 2 3	-148.0	155.5	90.0	0.0
dipolar 2 4	-19.1	164.6	90.0	0.0
dipolar 2 5	-7.8	-179.0	90.0	0.0
dipolar 2 6	-4.8	-161.6	90.0	0.0
dipolar 3 4	-140.0	173.4	90.0	0.0
dipolar 3 5	-23.8	-165.5	90.0	0.0
dipolar 3 6	-7.8	-146.5	90.0	0.0
dipolar 4 5	-167.1	-143.2	90.0	0.0
dipolar 4 6	-20.7	-126.9	90.0	0.0
dipolar 5 6	-125.73	-112.1	90.0	0.0

Table S9. Description of the spin systems used for SIMPSON simulations in Figure S13B.

Six-spin jR2R3-21Å	δ_{iso}	δ_{aniso}	η^{CS}	α_{PC}	β_{PC}	γ_{PC}
shift 1	0p	0	0	0	0	0
shift 2	1p	0	0	0	0	0
shift 3	3p	0	0	0	0	0
shift 4	-1p	0	0	0	0	0
shift 5	-3p	0	0	0	0	0
shift 6	-2.5p	0	0	0	0	0
	$b_{ij}/(2\pi)$	α_{PC}	β_{PC}	γ_{PC}		
dipolar 1 2	-178.2	90.0	90.0	0.0		
dipolar 1 3	-22.3	90.0	90.0	0.0		
dipolar 1 4	-1.6	25.6	90.0	0.0		
dipolar 1 5	-1.6	13.5	90.0	0.0		
dipolar 1 6	-3.2	0.0	90.0	0.0		
dipolar 2 3	-178.2	90.0	90.0	0.0		
dipolar 2 4	-1.6	13.5	90.0	0.0		
dipolar 2 5	-3.2	0.0	90.0	0.0		
dipolar 2 6	-1.6	-13.5	90.0	0.0		
dipolar 3 4	-3.2	0.0	90.0	0.0		
dipolar 3 5	-1.6	-13.5	90.0	0.0		
dipolar 3 6	-1.6	-25.6	90.0	0.0		
dipolar 4 5	-178.2	-90.0	90.0	0.0		
dipolar 4 6	-22.3	-90.0	90.0	0.0		
dipolar 5 6	-178.2	-90.0	90.0	0.0		

Table S10. Chemical shifts and line widths for the HSQC spectra of jR2R3 and S305^{p}

jR2R3-P301L	^{1}H (ppm)	^{15}N (ppm)	^{1}H LW (Hz)	^{15}N LW (Hz)
L301	8.428	126.42	17.35	20.58
V309	8.143	125.60	17.52	17.84
I308	8.227	124.08	15.75	15.69
V300	8.191	122.03	15.37	18.58
V306	8.101	121.54	16.46	16.33
I297	8.079	121.40	18.05	16.04
V313	8.150	120.69	14.44	15.40
**S305^{p}**	**^{1}H (ppm)**	**^{15}N (ppm)**	**^{1}H LW (Hz)**	**^{15}N LW (Hz)**
L301	8.44 (+0.01)	126.11 (-0.33)	15.99	15.35
V309	8.14 (0)	125.09 (-0.53)	17.58	15.25
I308	8.25 (+0.02)	123.72 (-0.37)	16.18	15.16
V300	8.22 (+0.03)	122.27 (+0.23)	18.28	16.31
V306	8.19 (+0.09)	122.00 (+0.45)	16.51	15.19
I297	8.17 (+0.09)	121.73 (+0.33)	15.99	15.99
V313	8.17 (+0.02)	120.8 (+0.1)	15.59	16.48

SR2$^1_8$ DQ-SQ SIMPSON script for a model system of ^{31}P spin pair of 1500 Hz dipolar coupling

----

```
spinsys {
  channels 31P
  nuclei 31P 31P
 shift 1 1p              0       0       0       0       0
 shift 2 10p             0       0       0       0       0
  dipole 1 2 -1500 0 0 0
}

par {
  variable       nu    1
  variable       small_n    8
  variable       large_N    2

  proton_frequency       500e6
  spin_rate              10000
  sw                      spin_rate
  np                     20
  method                 direct
  crystal_file           rep20
  gamma_angles           10
  start_operator         Inz
  detect_operator        I2p
  verbose                1101
  conjugate_fid          false

  variable       rf             spin_rate*0.5
  variable       t90            0.25e6/rf
  variable       t180           0.50e6/rf
  variable       t270           0.75e6/rf
  variable       t360            1.00e6/rf
}

proc pulseq {} {
  global par
```

```
 maxdt 1.0

 matrix set 1 totalcoherence {-2 +2}
 matrix set 2 totalcoherence {0}

reset

           pulseid 1 100000 x

           pulse $par(t360) $par(rf) 270
           pulse $par(t270) $par(rf) 90
           pulse $par(t90) $par(rf)  270

           pulse $par(t360) $par(rf) 90
           pulse $par(t270) $par(rf) 270
           pulse $par(t90) $par(rf)  90

            pulse $par(t360) $par(rf) 90
           pulse $par(t270) $par(rf) 270
            pulse $par(t90) $par(rf)  90

           pulse $par(t360) $par(rf) 270
           pulse $par(t270) $par(rf) 90
           pulse $par(t90) $par(rf)  270
 store 1

reset
           pulse $par(t360) $par(rf) 360
           pulse $par(t270) $par(rf) 180
           pulse $par(t90) $par(rf)  360

           pulse $par(t360) $par(rf) 180
           pulse $par(t270) $par(rf) 360
           pulse $par(t90) $par(rf)  180

           pulse $par(t360) $par(rf) 180
           pulse $par(t270) $par(rf) 360
```

```
            pulse $par(t90) $par(rf)  180

            pulse $par(t360) $par(rf) 360
            pulse $par(t270) $par(rf) 180
            pulse $par(t90) $par(rf)  360

            pulseid 1 100000 -x
store 2

       reset
       acq

  for {set i 1} {$i < $par(np)} {incr i} {
       reset
       prop 1 $i
       filter 1
       prop 2 $i
       filter 2
       pulseid $par(t90) $par(rf) y
       acq
      }
}

proc main {} {
global par

  fsave [fsimpson] $par(name).fid
}
```

SI References

[1] M. S. Nowotarski, L. R. Potnuru, J. S. Straub, R. Chaklashiya, T. Shimasaki, B. Pahari, H. Coffaro, S. Jain, S. Han, "Dynamic Nuclear Polarization Enhanced Multiple-Quantum Spin Counting of Molecular Assemblies in Vitrified Solutions" *J. Phys. Chem. Lett.* **2024**, 7084–7094.

[2] S. Sturniolo, T. F. G. Green, R. M. Hanson, M. Zilka, K. Refson, P. Hodgkinson, S. P. Brown, J. R. Yates, "Visualization and processing of computed solid-state NMR parameters: MagresView and MagresPython" *Solid State Nuclear Magnetic Resonance* **2016**, *78*, 64–70.

[3] S.-J. Park, N. Kern, T. Brown, J. Lee, W. Im, “CHARMM-GUI PDB Manipulator: Various PDB Structural Modifications for Biomolecular Modeling and Simulation” *Journal of Molecular Biology* **2023**, *435*, 167995.

[4] Lindahl, Abraham, Hess, Van Der Spoel **2022**, DOI 10.5281/ZENODO.5850051.

[5] S. Jo, T. Kim, V. G. Iyer, W. Im, “CHARMM-GUI: A web-based graphical user interface for CHARMM” *J Comput Chem* **2008**, *29*, 1859–1865.

[6] G. Bussi, D. Donadio, M. Parrinello, “Canonical sampling through velocity rescaling” *The Journal of Chemical Physics* **2007**, *126*, DOI 10.1063/1.2408420.

[7] H. J. C. Berendsen, J. P. M. Postma, W. F. Van Gunsteren, A. DiNola, J. R. Haak, “Molecular dynamics with coupling to an external bath” *The Journal of Chemical Physics* **1984**, *81*, 3684–3690.

[8] S. Nosé, “A molecular dynamics method for simulations in the canonical ensemble” *Molecular Physics* **1984**, *52*, 255–268.

[9] W. G. Hoover, “Canonical dynamics: Equilibrium phase-space distributions” *Phys. Rev. A* **1985**, *31*, 1695–1697.

[10] M. Parrinello, A. Rahman, “Polymorphic transitions in single crystals: A new molecular dynamics method” *Journal of Applied Physics* **1981**, *52*, 7182–7190.

[11] N. Michaud-Agrawal, E. J. Denning, T. B. Woolf, O. Beckstein, “MDAnalysis: A toolkit for the analysis of molecular dynamics simulations” *J Comput Chem* **2011**, *32*, 2319–2327.

[12] A. V. Martinez, L. Dominguez, E. Małolepsza, A. Moser, Z. Ziegler, J. E. Straub, “Probing the Structure and Dynamics of Confined Water in AOT Reverse Micelles” *J. Phys. Chem. B* **2013**, *117*, 7345–7351.

[13] P. Smith, R. M. Ziolek, E. Gazzarrini, D. M. Owen, C. D. Lorenz, “On the interaction of hyaluronic acid with synovial fluid lipid membranes” *Phys. Chem. Chem. Phys.* **2019**, *21*, 9845–9857.

[14] R. J. Gowers, P. Carbone, “A multiscale approach to model hydrogen bonding: The case of polyamide” *The Journal of Chemical Physics* **2015**, *142*, 224907.

[15] D. Massiot, F. Fayon, M. Capron, I. King, S. Le Calvé, B. Alonso, J.-O. Durand, B. Bujoli, Z. Gan, G. Hoatson, “Modelling one- and two-dimensional solid-state NMR spectra: Modelling 1D and 2D solid-state NMR spectra” *Magn. Reson. Chem.* **2002**, *40*, 70–76.

[16] M. P. Vigers, S. Lobo, S. Najafi, A. Dubose, K. Tsay, P. Ganguly, A. P. Longhini, Y. Jin, S. K. Buratto, K. S. Kosik, M. S. Shell, J.-E. Shea, S. Han, **2023**, DOI: 10.1101/2023.11.28.568818.

[17] M. P. Vigers, S. Lobo, S. Najafi, A. Dubose, K. Tsay, P. Ganguly, A. P. Longhini, Y. Jin, S. K. Buratto, K. S. Kosik, M. S. Shell, J.-E. Shea, S. Han, “Water-directed pinning is key to tau prion formation” *Proc. Natl. Acad. Sci. U.S.A.* **2025**, *122*, e2421391122.

[18] T. Arakhamia, C. E. Lee, Y. Carlomagno, M. Kumar, D. M. Duong, H. Wesseling, S. R. Kundinger, K. Wang, D. Williams, M. DeTure, D. W. Dickson, C. N. Cook, N. T. Seyfried, L. Petrucelli, J. A. Steen, A. W. P. Fitzpatrick, “Posttranslational Modifications Mediate the Structural Diversity of Tauopathy Strains” *Cell* **2020**, *180*, 633-644.e12.

[19] Y. Shi, W. Zhang, Y. Yang, A. G. Murzin, B. Falcon, A. Kotecha, M. Van Beers, A. Tarutani, F. Kametani, H. J. Garringer, R. Vidal, G. I. Hallinan, T. Lashley, Y. Saito, S. Murayama, M. Yoshida, H. Tanaka, A. Kakita, T. Ikeuchi, A. C. Robinson, D. M. A. Mann, G. G. Kovacs, T. Revesz, B. Ghetti, M. Hasegawa, M. Goedert, S. H. W. Scheres, “Structure-based classification of tauopathies” *Nature* **2021**, *598*, 359–363.
